# Five-year costs from a randomised comparison of bilateral and single internal thoracic artery grafts

**DOI:** 10.1136/heartjnl-2018-313932

**Published:** 2019-04-04

**Authors:** Matthew Little, Alastair Gray, Doug Altman, Umberto Benedetto, Marcus Flather, Stephen Gerry, Belinda Lees, Jacqueline Murphy, Helen Campbell, David Taggart

**Affiliations:** 1 Nuffield Department of Population Health, University of Oxford, Oxford, UK; 2 Nuffield Department of Population Health, University of Oxford Health Economics Research Centre, Oxford, UK; 3 Nuffield Department of Orthopaedics, Rheumatology and Musculoskeletal Sciences, Centre for Statistics in Medicine, University of Oxford, Oxford, UK; 4 University of Bristol School of Clinical Science, Bristol, Bristol, UK; 5 University of East Anglia Faculty of Medicine and Health Sciences, Norwich, Norfolk, UK; 6 Norfolk and Norwich University Hospitals NHS Foundation Trust, Norwich, Norfolk, UK; 7 Nuffield Department of Orthopaedics, Rheumatology and Musculoskeletal Sciences, Centre for Statistics in Medicine, Oxford, UK; 8 Nuffield Department of Surgical Sciences, John Radcliffe Hospital, Oxford, Oxfordshire, UK

**Keywords:** health care economics, coronary artery disease, coronary artery disease surgery

## Abstract

**Background:**

The use of bilateral internal thoracic arteries (BITA) for coronary artery bypass grafting (CABG) may improve survival compared with CABG using single internal thoracic arteries (SITA). We assessed the long-term costs of BITA compared with SITA.

**Methods:**

Between June 2004 and December 2007, 3102 patients from 28 hospitals in seven countries were randomised to CABG surgery using BITA (n=1548) or SITA (n=1554). Detailed resource use data were collected from the initial hospital episode and annually up to 5 years. The associated costs of this resource use were assessed from a UK perspective with 5 year totals calculated for each trial arm and pre-selected patient subgroups.

**Results:**

Total costs increased by approximately £1000 annually in each arm, with no significant annual difference between trial arms. Cumulative costs per patient at 5-year follow-up remained significantly higher in the BITA group (£18 629) compared with the SITA group (£17 480; mean cost difference £1149, 95% CI £330 to £1968, p=0.006) due to the higher costs of the initial procedure. There were no significant differences between the trial arms in the cost associated with healthcare contacts, medication use or serious adverse events.

**Conclusions:**

Higher index costs for BITA were still present at 5-year follow-up mainly driven by the higher initial cost with no subsequent difference emerging between 1 year and 5 years of follow-up. The overall cost-effectiveness of the two procedures, to be assessed at the primary endpoint of the 10-year follow-up, will depend on composite differences in costs and quality-adjusted survival.

**Trial registration number:**

ISRCTN46552265

## Introduction

Coronary artery bypass grafting (CABG) is one of the most commonly performed operations globally and an established and effective treatment for symptomatic multivessel coronary artery disease.^1^ The routine surgical practice has been to graft a single internal thoracic artery (SITA) to the left anterior descending coronary artery and the use of vein or radial-artery grafts to bypass other coronary arteries.^2^ The excellent outcomes of SITA have stimulated interest in the use of bilateral internal thoracic arteries (BITA).^3^

Existing evidence from observational studies of the effect of BITA on long-term survival suggests that BITA is associated with a reduction in mortality compared with SITA. A recent meta-analysis of observational studies from nine eligible studies including 15 583 patients with mean follow-up exceeding 9 years estimated a Hazard Ratio (HR) of 0.79 (95% CI 0.75 to 0.84) for BITA compared with SITA.^4^ However, BITA has not been widely adopted due to it being a more complex procedure, associated with a higher risk of sternal wound complications and a lack of randomised evidence of benefit.

The Arterial Revascularisation Trial (ART) was designed to address these concerns, with a primary objective of comparing 10-year survival rates associated with BITA over SITA. ART has reported clinical and safety outcomes across trial arms at 5 years postrandomisation.^5^ CABG is a high-volume procedure, with approximately 20 000 carried out in England every year; therefore, it is important to consider the long-term impact on costs as well as clinical effectiveness. ART was designed with an integrated health economic evaluation and will ultimately report on the cost-effectiveness of BITA versus SITA at 10 years. A 1-year cost comparison has previously been published, showing BITA to be associated with 9% higher costs, primarily due to longer time in theatre and in-hospital stay, and slightly higher costs related to sternal wound problems during follow-up.^6^ However, it is possible that these differences are offset in the long run. This paper investigates this possibility by providing a comparison of resource use and costs up to 5 years postrandomisation.

## Method

Details of the ART protocol, baseline data, 1-year safety outcomes and 5-year clinical and safety outcomes have been published previously.^5 7^ ART is a multicentre randomised control trial involving 28 hospitals across seven countries with a primary outcome of all-cause mortality at 10 years of follow-up. The trial complied with the Declaration of Helsinki. Prior ethics approval was obtained at all the participating centres and each patient was required to provide written informed consent.

Patients were eligible for the trial if they had multivessel coronary artery disease and were scheduled to undergo CABG as part of their routine care plan (this included patients requiring urgent surgery, but not those with evolving myocardial infarction). Patients requiring only single grafts or concomitant valve surgery, as well as those with a history of CABG, were excluded. Patients were followed up at a routine clinical visit 6 weeks postsurgery and then annually by telephone call and postal questionnaires.

### Measurement of resource use

The cost analysis of the two interventional strategies followed the general methods published previously but extended the analysis to assess resource costs at the 5-year follow-up on all patients. Information was collected at each annual follow-up on medication use, subsequent sternal wound complications, serious adverse events, the frequency of visits to a general practitioner (GP), practice nurse, hospital outpatient clinic or cardiac rehabilitation clinic, and duration of any hospital readmission.

### Measurement of costs

Costs were evaluated from the perspective of the English National Health Service (NHS). Clinical events and resource use during the initial hospital stay were costed using sources, methods and assumptions published previously,^6^ with all costs updated to 2016/2017 prices using the hospital and community health services index.^8^ All resources used over the remaining 5 years of follow-up were costed using the appropriate 2016/2017 unit costs.

Details of the costing methodology can be found in the online supplementary material of the 1-year analysis.^6^ Following this methodology, GP and practice nurse visits were costed using Personal Social Services Research Unit estimates applied to all reported visits, and NHS reference costs provided unit costs for all recorded hospital outpatient clinic and cardiac rehabilitation clinic visits. Out-of-pocket costs, such as travel costs and time spent on GP visits, were not collected as the perspective of the analysis was the healthcare system. Costs associated with the clinical events of myocardial infarction, cerebrovascular accidents, further CABG, percutaneous coronary interventions (PCI) or cardiac catheterisations were obtained from appropriate 2016/2017 NHS Reference Costs. Reference costs were adjusted for clinical events occurring during the index admission to avoid double counting. The most frequent diagnosis groups classified as ‘other’ included musculoskeletal, gastrointestinal, cardiac arrhythmias and genitourinary. The cost impact of these events was assumed to be captured by costing the length of stay of the admission. An emergency department attendance was assumed where participants were admitted for an event, but no overnight stay was reported. Individual drug usage was costed using unit costs from the NHS electronic Market Information Tool. Full details of the sources and assumptions used in the costings can be found in the online supplementary table 1.

10.1136/heartjnl-2018-313932.supp1 Supplementary file 1

### Missing data

Some items of data were missing as a result of incomplete responses or loss to follow-up. Descriptive analysis revealed that 70% of observations in each arm of the trial provided complete data for all resources used across the follow-up period. Logit models of missingness on baseline variables indicated that having some missing data was statistically significantly associated with baseline hospital, smoking status and sex. This suggests that the data are not missing completely at random as is assumed in complete case analysis. Therefore, multiple imputation was used to impute missing resource use in the trial data to limit the loss of power and bias arising from the exclusion of missing data; unit costs were then attached to the imputed resource use data.

Imputation was implemented separately by randomised treatment allocation. Missing data were imputed at the most disaggregated level at which the model would converge. Chained equations using predictive mean matching or logistic regression were used to impute missing values for each variable.^9^ Imputation was conducted using the baseline hospital, age, sex, baseline Canadian Cardiovascular Society (CCS) class, diabetes, smoking status and peripheral arterial disease. Following the rule of thumb that the number of imputations should be at least equal to the percentage of incomplete cases (30%),^9^ the procedure was repeated 30 times to produce 30 imputed datasets with Rubin’s Rule used to summarise across imputations.^10^

Statistical analysis of imputed data summarised continuous data using means and categorical data using percentages. In line with recommended practice in cost analyses,^11^ we report mean costs, but also report median values for total costs in the online supplementary table 11. Two-sample t-tests were used for comparisons of mean differences and 95% CIs for differences were calculated. Standard errors were adjusted to account for clustering at the hospital level. All data analyses were performed using STATA 14.

### Sensitivity analyses

The robustness of estimates to the imputation of missing data was explored by analysing the costs of a complete case sample. In addition, the robustness of estimates including only patients who received the surgery they were allocated was investigated. Uncertainty surrounding individual unit costs is not reported, as the low and multiple unit costs and infrequency of many clinical events meant that extreme changes in assumptions were required to produce even modest effects on results.

### Subgroup analyses

Total annual cost at each time point and total cumulative costs were compared between BITA and SITA arms for selected pre-specified subgroups. These were: insulin-dependent diabetic, non-insulin dependent diabetic and non-diabetic, age ≥70 years versus <70 years, on-pump versus off-pump, prior myocardial infarction versus no prior myocardial infarction, New York Heart Association (NYHA) class I and II versus NYHA class III and IV, and CCS class 0, I and II versus CCS class III and IV. Five-year costs were also compared in each of the three countries (UK, Poland and Australia) which recruited >100 patients to the trial.

## Results

Table 1 shows annual resource use and the frequency of adverse events by the two trial arms. Similar levels of resource use and counts of adverse events were observed in each trial arm at each time point. The two trial arms had similar frequencies of GP visits, nurse visits, outpatient visits, cardiac rehabilitation visits and nights in hospital at each time point. This resulted in there being no significant differences in cumulative healthcare contacts across 5 years of follow-up. Similar proportions of participants experienced an adverse event in each trial arm for the counts of myocardial infarction, cerebrovascular accident, major bleeds and deaths from any cause, whereas significantly more sternal wound problems and ‘other’ adverse events were reported in the BITA arm across 5 years of follow-up.

**Table 1:** Follow-up mean resource use per patient and frequency of adverse events

	Year 1		Year 2		Year 3		Year 4		Year 5		Total at year 5		Difference (95% CI to P values)
	Mean resource use/number of patients receiving resources												
	SITA (n=1554)	BITA (n=1548)	SITA (n=1554)	BITA (n=1548)	SITA (n=1554)	BITA (n=1548)	SITA (n=1554)	BITA (n=1548)	SITA (n=1554)	BITA (n=1548)	SITA (n=1554)	BITA (n=1548)	BITA vs SITA
Healthcare contacts													
GP visits	6.5	6.3	4.5	4.4	4.2	4.1	4.1	3.8	3.9	3.8	20.8	20.0	−0.8 (−1.9 to 0.4; 0.195)
Nurse visits	3.2	3.4	1.2	1.4	1.4	1.4	1.4	1.5	1.4	1.4	7.8	8.5	0.7 (−0.5 to 1.8; 0.266)
Outpatient clinic visits	1.9	2.4	1.2	1.5	1.0	1.3	1.2	1.1	1.1	1.1	5.9	6.8	0.9 (−0.1 to 1.9; 0.089)
Cardiac rehabilitation visits	5.1	5.2	1.2	1.1	0.9	0.7	0.7	1.0	0.5	0.5	8.1	8.0	−0.1 (−1.7 to 1.6; 0.953)
Number of nights in hospital	0.7	1.0	0.2	0.1	0.1	0.2	0.1	0.1	0.2	0.1	1.1	1.3	0.2 (−0.3 to 0.7; 0.362)
Medications													
Mean number of medications	4.8	4.8	4.7	4.7	4.6	4.7	4.6	4.5	4.6	4.5	21.1	20.8	−0.3 (−0.9 to 0.3; 0.328)
Adverse events to n (%)													
Myocardial infarction	12	9	2	5	4	8	5	6	7	6	30 (0.39)	34 (0.44)	−0.05 (−0.3 to 0.1; 0.606)
Cerebrovascular accident	14	9	5	6	7	3	3	5	10	8	39 (0.50)	31 (0.40)	0.10 (−0.1 to 0.3; 0.360)
Further CABG	0	2	0	0	0	1	0	0	0	0	0 (0.00)	3 (0.04)	−0.04 (−0.1 to 0.0; 0.179)
Further PCI	25	24	26	13	26	21	11	18	17	17	105 (1.35)	93 (1.20)	0.15 (−0.3 to 0.6; 0.476)
Revascularisation with catheter	3	12	17	13	23	10	14	9	3	12	60 (0.77)	56 (0.72)	0.05 (−0.3 to 0.4; 0.773)
Sternal wound problems	34	67	3	1	0	0	1	2	0	0	38 (0.49)	70 (0.90)	−0.42 (−0.8 to –0.1; 0.017)
Major bleed	5	6	0	0	0	0	0	1	0	0	5 (0.06)	7 (0.09)	−0.03 (−0.1 to 0.1; 0.559)
Other events	396	376	146	185	136	172	140	168	109	147	927 (11.9)	1048 (13.5)	−1.61 (−3.0 to –0.2; 0.028)
Death	25	21	25	18	23	33	26	28	29	23	128 (1.65)	123 (1.59)	0.06 (−0.3 to 0.5; 0.774)

SITA, single internal thoracic artery; BITA, bilateral internal thoracic artery; CABG, coronary artery bypass graft surgery; GP, general practitioner; PCI, percutaneous coronary interventions.

Table 2 shows the corresponding mean costs of resource use in each trial arm and by year of follow-up. No significant difference was observed for the cost of visits or hospitalisations (in total or by sub-category), or total medication usage. The only adverse events with a significant difference in costs were sternal wound problems.

**Table 2:** Follow-up mean costs (£) per patient by trial arm

	Year 1 Mean cost		Year 2 Mean cost		Year 3 Mean cost		Year 4 Mean cost		Year 5 Mean cost		Total at year 5		Difference (95% CI to P values)
	SITA (n=1554)	BITA (n=1548)	SITA (n=1554)	BITA (n=1548)	SITA (n=1554)	BITA (n=1548)	SITA (n=1554)	BITA (n=1548)	SITA (n=1554)	BITA (n=1548)	SITA (n=1554)	BITA (n=1548)	BITA vs SITA
Healthcare contacts													
GP visits	240	234	166	162	156	153	150	142	143	140	855	831	−23.7 (−70 to 23; 0.319)
Nurse visits	46	50	17	20	20	21	20	22	20	21	122	133	11.3 (−6 to 29; 0.203)
Outpatient clinic visits	247	309	153	187	124	167	158	148	142	145	822	955	133.2 (−7 to 273; 0.062)
Cardiac rehabilitation visits	370	378	84	82	63	50	49	70	39	39	606	619	13.3 (−112 to 139; 0.835)
Number of nights in hospital	168	234	87	53	44	81	67	64	106	76	472	509	36.8 (−94 to 167; 0.581)
All healthcare contacts	1071	1205	506	504	407	473	444	446	450	422	2877	3048	170.9 (−78 to 420; 0.178)
Medications													
Total medication	37	38	39	41	40	44	41	45	41	43	198	211	13.0 (−9 to 34; 0.237)
Adverse event treatment													
Myocardial infarction	15	12	3	5	6	9	7	6	9	9	40	41	1.5 (−21 to 24; 0.897)
Cerebrovascular accident	26	13	12	12	10	7	7	10	21	16	76	57	−18.1 (−52 to 16; 0.300)
Further CABG	0	6	0	0	0	6	0	0	0	0	0	13	12.7 (−5 to 30; 0.156)
Further PCI	54	41	43	24	41	41	17	32	34	29	189	167	−22.0 (−86 to 42; 0.501)
Revascularisation with catheter	4	18	16	20	27	14	18	11	2	18	67	79	12.7 (−22 to 47; 0.471)
Sternal wound problems	104	295	1	0	0	0	0	1	0	0	105	296	191.1 (66 to 317; 0.003)
Major bleed	58	83	0	0	0	0	0	1	0	0	58	84	26.1 (−64 to 116; 0.570)
Other events (cost of hospital stay only)	459	500	191	170	168	152	161	189	225	181	1204	1192	−12.1 (−344 to 320; 0.943)
Death (cost of hospital stay only)	58	26	34	19	5	40	55	10	30	34	182	128	−53.7 (−168 to 60; 0.357)
All adverse event costs	777	994	299	249	258	269	266	259	320	286	1920	2058	138.3 (−283 to 559; 0.520)
All costs	1848	2199	805	753	664	741	709	705	770	708	4995	5317	322.1 (−207 to 852; 0.233)
Difference in costs (p value)	351 (0.028)		−52 (0.469)		77 (0.259)		−4 (0.952)		−63 (0.561)				

SITA, single internal thoracic artery; BITA, bilateral internal thoracic artery; CABG, coronary artery bypass graft surgery; GP, general practitioner; PCI, percutaneous coronary interventions.

**Figure 1:**
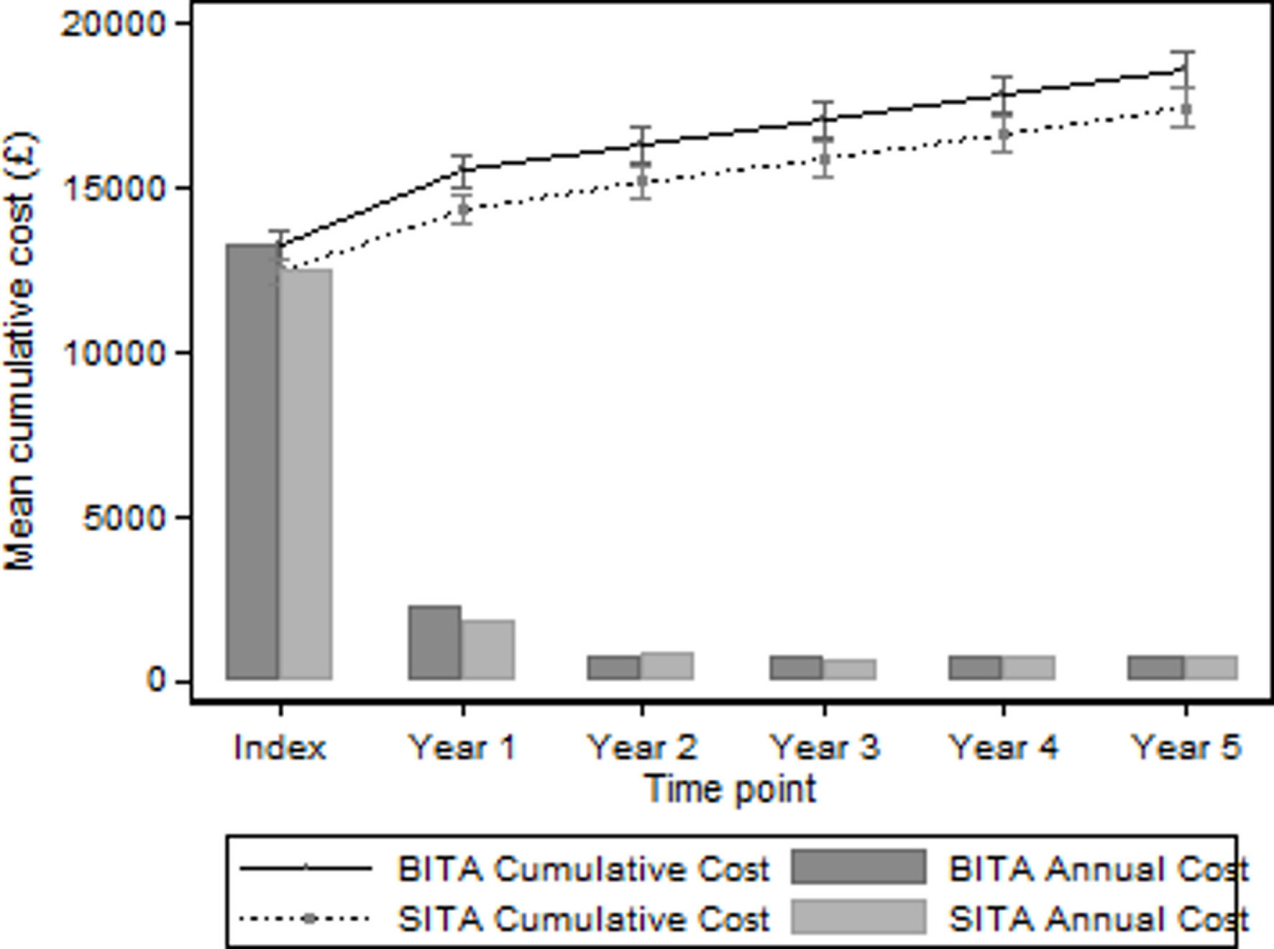
Mean cumulative total cost and mean annual follow-up costs.

Table 3 and figure 1 show the mean cumulative costs for each year and each trial arm, and the mean difference. The index hospitalisation had a total cost of £12 485 in the SITA group compared with £13 312 in the BITA group with a significant difference of £827 (95% CI £261 to £1392, p<0.004). Annual costs increased in each arm by approximately £1000 annually, with no evidence of the mean difference that had emerged by the end of year one changing significantly over time. By year 5, cumulative costs were on average £1149 higher in the BITA group than the SITA group (95% CI £330 to £1968, p<0.006). This difference was mainly due to the higher cost of the initial procedure.

**Table 3:** Mean cumulative total costs (£) from index admission to the 5-year follow-up

	SITA (n=1554)	BITA (n=1548)	BITA vs SITA
Index	12 485	13 312	827 (261 to 1392; 0.004)
Year 1	14 370	15 548	1178 (503 to 1854; 0.001)
Year 2	15 214	16 342	1128 (419 to 1837; 0.002)
Year 3	15 919	17 127	1209 (471 to 1947; 0.001)
Year 4	16 669	17 878	1209 (436 to 1981; 0.002)
Year 5	17 480	18 629	1149 (330 to 1968; 0.006)

SITA, single internal thoracic artery; BITA, bilateral internal thoracic artery.

**Figure 2:**
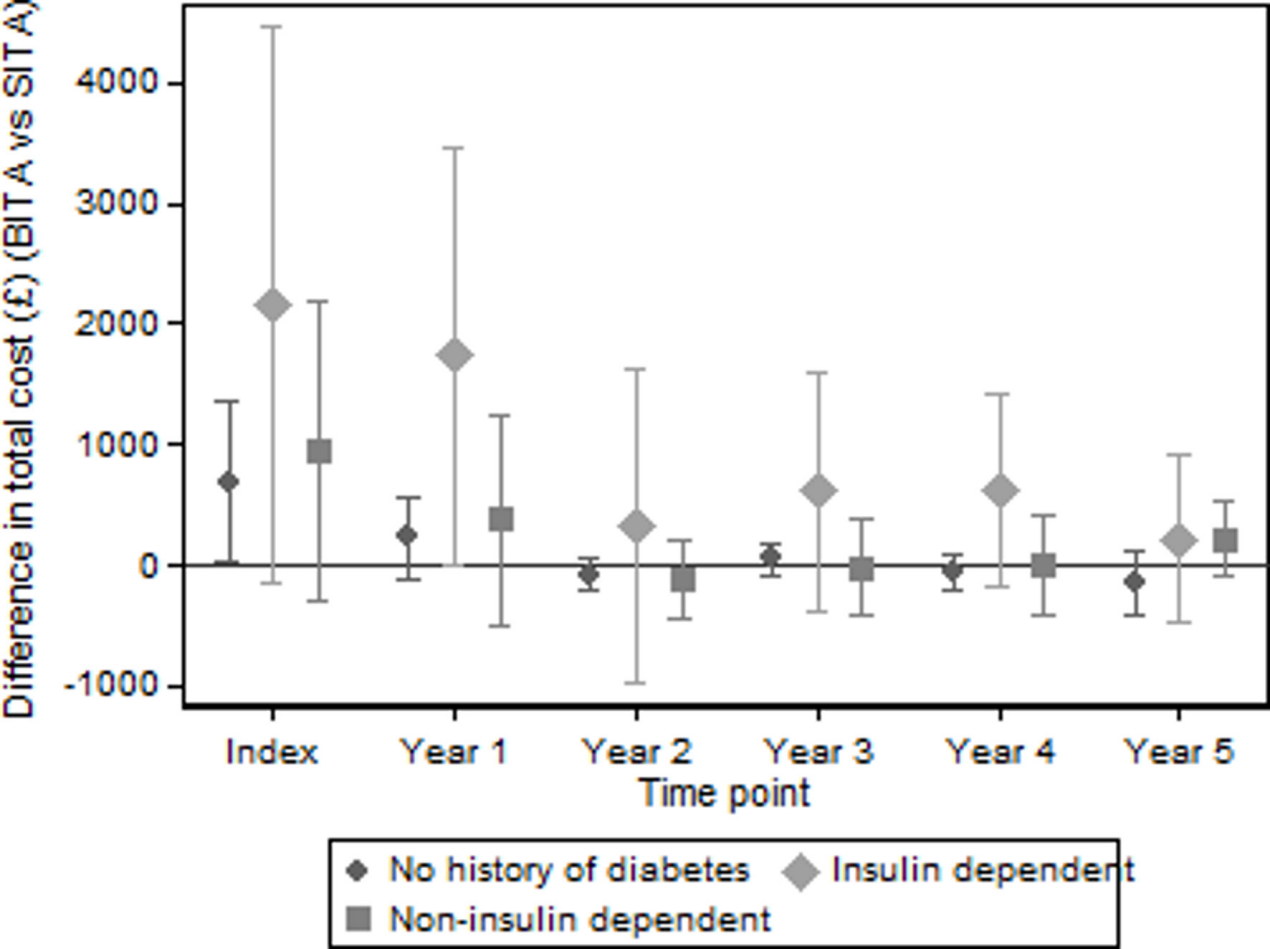
Differences in mean total costs (BITA vs SITA, 95% CI) by baseline history of diabetes over time. BITA, bilateral internal thoracic artery; SITA, single internal thoracic artery.

Table 4 shows total costs to the 5-year follow-up for the various prespecified subgroup analyses and the country comparisons. The difference between the trial arms was found to vary by subgroup, with the largest differences being observed when separating patients by baseline diabetes history. Compared with the cumulative mean difference in total costs of £1149 across all patients, mean additional costs in the BITA arm were £5673 higher (95% CI £1334 to £10 012, p<0.011) for patients who were insulin-dependent, but non-significantly higher (£681, 95% CI -£227 to £1590, p<0.142) for patients who were without diabetes. Figure 2 shows how these differences evolved over the first 5 years of the trial. Table 4 also shows that differences in costs between the trial arms were somewhat larger for patients with a history of myocardial infarction, more severe angina or cardiac disease compared with those with no history, for on-pump compared with off-pump patients, for CCS class III and above compared with class 0, I and II, and for patients in Australia. Full details of the difference between trial arms by subgroup are provided in the online supplementary tables 4–10 and figures 1–6.

**Table 4:** Total costs (£) to the 5-year follow-up by trial arm and by subgroups

	SITA Mean cost	BITA Mean cost	BITA vs SITA Mean difference (95% CI to P values)
No history of diabetes (n=2368)	17 269	17 951	681 (−227 to 1590; 0.142)
Insulin-dependent diabetes (n=174)	18 355	24 028	5673 (1334 to 10012; 0.011)
Non-insulin-dependent diabetes (n=560)	17 957	19 403	1447 (−474 to 3367; 0.140)
Aged <70 years (n=2271)	16 474	17 842	1368 (538 to 2198; 0.001)
Aged ≥70 years (n=831)	20 042	20 666	623 (−1398 to 2644; 0.545)
Off-pump (n=1259)	17 905	18 065	160 (−1237 to 1557; 0.823)
On-pump (n=1819)	17 256	19 214	1958 (985 to 2931; 0.000)
No prior MI (n=1800)	17 222	18 088	866 (−150 to 1882; 0.095)
Prior MI (n=1300)	17 746	19 260	1514 (187 to 2840; 0.025)
NYHA class I and II (n=2431)	17 556	18 296	740 (−132 to 1612; 0.096)
NYHA class III and IV (n=669)	17 039	19 582	2542 (534 to 4550; 0.013)
CCS class 0 to I, II (n=2143)	17 571	18 185	614 (−347 to 1575; 0.210)
CCS class III to IVa/b/c (n=959)	17 175	19 452	2278 (769 to 3786; 0.003)
UK (n=2053)	18 052	18 864	813 (−306 to 1932; 0.154)
Poland (n=606)	15 691	16 811	1120 (130 to 2109; 0.027)
Australia (n=192)	19 878	21 767	1889 (−682 to 4460; 0.149)

BITA, bilateral internal thoracic artery; CCS, Canadian Cardiovascular Society; MI, myocardial infarction NYHA, New York Heart Association; SITA, single internal thoracic artery.

## Discussion

ART is the first and largest randomised comparison of SITA and BITA ever conducted and has permitted the first detailed comparison of costs of these procedures over a 5-year period. We found the significantly higher index cost of BITA (£827, 95% CI £261 to £1392) is maintained up to the 5-year follow-up as a result of similar total costs observed in each arm over each subsequent follow-up period.

This is the first study to compare the cost of BITA and SITA at 5 years postsurgery. The finding of similar total costs following the initial procedure over a 5-year period is in contrast to previous studies comparing the cost-effectiveness of CABG and PCI, which have found differences in costs to emerge over a similar length of time. Evidence from the SYNTAX trial, for example, found that an initial difference of about $10 000 post index admission reduced to $5600 after 5 years of follow-up due lower revascularisation rates and medication usage in the CABG group.^12^ It is possible that differences between BITA and SITA may eventually emerge due to the superior long-term graft patency achieved with arterial grafts.

Higher cumulative costs of BITA compared with SITA at 5 years were found to be particularly marked in patients who were insulin-dependent compared with those who were non-insulin dependent or were without diabetes. The online supplementary table 4 shows that this difference was primarily driven by higher outpatient costs, longer stays in hospital and costs associated with sternal wound problems, which are well recognised to be more frequent in patients with diabetes. Indeed, several observational studies have found diabetes and BITA to be independent risk factors for sternal wound infections following CABG.^13 14^ However, a recent observational study found BITA to only be an independent predictor in patients with chronic complications of diabetes mellitus.^15^

Differences in costs also varied by surgical technique, with higher mean total costs observed for BITA patients who underwent on-pump CABG while no significant difference was observed for patients who underwent off-pump CABG. This difference in costs was primarily driven by the higher cost of the index admission of BITA patients who underwent on-pump CABG. This finding compliments those of CORONARY (the CABG off or On Pump Revascularization Study), which showed no significant difference in 5-year total costs (mean difference $115, 95% CI -$697 to $927) between patients who underwent off-pump or on-pump CABG.^16^

The analysis in this paper is based on the randomised comparison of BITA and SITA ever conducted, which reduces the potential of bias arising from unobserved factors. Our findings are in contrast to a recent retrospective study comparing BITA and SITA using a large US observational sample, which found lower costs for BITA and a shorter length of stay during the index admission.^15^ These differences may be the result of bias arising from unobserved heterogeneity between patients in the non-randomised study.

The analysis in this paper assumed missing data to be MAR with predictions based on the resource use of similar patients without missing data. It is not possible to validate this assumption; however, sensitivity analysis can explore how the results are affected if data were assumed to be missing not at random. Following the recommendation of Faria *et al*,^17^ this was achieved by shifting imputed data by a sensitivity parameter to give a dataset imputed under MNAR. The included values of the sensitivity parameter varied imputed costs between −30% and 30% at 5% point intervals. The results from this analysis are shown in the online supplementary figure 9, which found that the difference in mean cost at the 5-year follow-up varied between £950 and £1350.

A potential limitation of the current analysis was the application of UK-based unit costs to resource use from seven countries. This could systematically misestimate total costs if differences in relative prices between countries have resulted in systematically different patterns of resource use. However, such an effect would not necessarily bias the randomised comparison. The data and results presented here should permit analysts to conduct analyses from the perspective of countries other than the UK using appropriate local unit cost sets.

## Conclusion

The higher initial costs of BITA compared with SITA were still present at the 5-year follow-up, with similar levels of resource use each year following the index procedure. Other differences may emerge by the time all patients reach the 10-year follow-up, the relevant time-point for the primary outcome of the trial. Finally, in order to assess the cost-effectiveness of BITA versus SITA, any differences in cost will have to be viewed alongside any differences in quality-adjusted survival, which will be reported at the 10-year follow-up.

**Key message**

**What is already known on this subject?**

- The use of a single internal thoracic artery (SITA) for coronary artery bypass grafting is a safe, effective and high-volume procedure, but bilateral internal thoracic arteries (BITA) may offer improved long-term patient outcomes.

**What might this study add?**

- No randomised comparison of the long-term costs of these procedures has previously been published. Using data from the Arterial Revascularisation Trial, we showed that the higher index costs of BITA were still present at the 5-year follow-up mainly driven by the higher initial cost with no subsequent difference emerging between 1 year and 5 years of follow-up. Larger differences were observed in certain patient subgroups, particularly diabetes versus non-diabetes.

**How might this impact on clinical practice?**

- Clinicians and healthcare policy-makers will find our results of value when considering the potential cost implications of moving from SITA to BITA. These cost estimates will also be required for researchers to assess the long-term cost-effectiveness of BITA.
