# Supplementary material for: Five-year costs from a randomised comparison of bilateral and single internal thoracic artery grafts
Source: Heart. 2019 Apr 4;105(16):1237–43. doi: 10.1136/heartjnl-2018-313932 (PMC6678045; doi:10.1136/heartjnl-2018-313932)
Supplement: Supplementary file 1 [file heartjnl-2018-313932supp001.pdf]

S Table 1: Unit costs used to value resource use data collected from ART

Resource category	Unit cost (2016/17 GBP/£)	Assumptions and Source
Health care contact at follow-up		
**GP clinic attendance**	37.00	Unit Costs of Health and Social Care 2017 (section 10.3b). Per surgery consultation lasting 9.22 minutes.
**Visits to practice nurse**	14.47	Unit Costs of Health and Social Care 2017. Per patient contact lasting 15.5 minutes
**Outpatient clinic attendance**	128.72	NHS Reference Costs – Total Outpatient Attendance - Cardiology outpatient clinic (320).
**Cardiac rehabilitation clinic attendance**	72.96	NHS Reference Costs – Total Outpatient Attendance - Cardiac rehabilitation outpatient clinic (327).
**Hospital re-admission bed day**	305.85	NHS Reference Costs – Index - Non-elective inpatient excess bed day cost. Weighted average of all admission types.
Medications at follow-up (per day)		
**Aspirin**	0.01	75mg per day for duration of follow-up.
**Clopidogrel**	0.02	75 mg per day for a period of three months unless otherwise stated.
**Warfarin**	0.02	6mg per day for duration of follow-up.
**Beta-blockers**	0.01	Assumed drug is Biprosolol at 5mg per day for duration of follow-up.
**Calcium-channel antagonists**	0.01	Assumed drug is Amlodipine at 5mg per day for duration of follow-up.
**Nitrates**	0.07	Assumed drug is Isosorbide Mononitrate at 40mg per day for duration of follow-up.
**Potassium channel activators**	0.13	Assumed drug is Nicorandil at 40mg per day for duration of follow-up.
**Statins**	0.03	Assumed half of patients received Simvastatin at 40mg per day and half Atorvastatin at 80mg per day for duration of follow-up.
**Other lipid lowering drugs**	0.00	Assumed drug is Ezetimibe at 10mg per day -up.
**ACE inhibitors**	0.02	Assumed drug is Ramipril at 5mg per day.
**Angiotensin-II antagonists**	0.03	Assumed half of patients received Losartan at 100mg per day and half Candesartan at 32mg per day for duration of follow-up.
**Diuretics**	0.00	Assumed drug is Furosemide at 40mg per day for a period of four weeks after surgery.
**Digoxin**	0.02	250mcg per day for duration of follow-up.
**Amiodarone**	0.11	600mg per day for duration of follow-up.
Adverse events at follow-up requiring admission		
**Myocardial infarction**	2213.04	NHS Reference Costs – Non-elective inpatient - Actual or Suspected Myocardial Infarction (weighted average across CC scores) (EB10).
**Cerebrovascular accident**	3558.94	NHS Reference Costs – Non-elective inpatient - Cerebrovascular Accident, Nervous System Infections or Encephalopathy (weighted average across CC scores) (AA22).
**Further CABG**	9804.89	NHS Reference Costs – Non-elective inpatient – Complex / Major / Standard Coronary Artery Bypass Graft (weighted average across CC scores) (ED26/7/8).
**Further PCI**	3655.15	NHS Reference Costs – Non-elective inpatient – Complex / Standard Percutaneous Transluminal Coronary Angioplasty (weighted average across CC scores) (EY40/1).
**Revascularisation with catheterisation only**	3582.59	NHS Reference Costs – Non-elective inpatient - Complex / Standard Cardiac Catheterisation Angioplasty (weighted average across CC scores) (EY42/3).
**Major bleed**	5953.62	Assumed to require surgery for investigation and to incur the same cost as sternal wound infection requiring reconstruction.
**Other adverse events**	Various	Costed by associated length of hospital stay using the cost of a non-elective inpatient excess bed day (£305.85 per day).
Adverse events at follow-up without admission		
**Myocardial infarction**	521.99	NHS Reference Costs - Day case - Actual or Suspected Myocardial Infarction, General day case (weighted average across CC scores) (EB10).
**Cerebrovascular accident**	561.75	NHS Reference Costs – Day case - Cerebrovascular Accident, Nervous System Infections or Encephalopathy (weighted average across CC scores) (AA22).
**Further PCI**	1980.06	NHS Reference Costs– Day case - Complex / Standard Percutaneous Transluminal Coronary Angioplasty (weighted average across CC scores) (EY40/1).
**Major bleed**	5953.62	Assumed to require surgery for investigation and to incur the same cost as sternal wound infection requiring reconstruction.
**Revascularisation with catheterisation only**	1001.19	NHS Reference Costs– Day case - Complex / Standard Cardiac Catheterisation (weighted average across CC scores) (EY42/3).

**Emergency investigations**	258.38	NHS Reference Costs– A&E category 4 or 5 treatment (weighted average). Used as an estimate for day hospital cost for patients not admitted.
Theatre Resource Use		
**Operating theatre time (per minute)**	24.74	Average of theatre running costs (inclusive of staff) divided by theatre hours for cardiology at 8 boards in Scotland carrying out CABG (cardiac surgery).
**Drugs for anaesthetic induction**	4.81	Assuming 1mg Fentanyl , 5mg Midazolam, 1 amp (4mg) Pancuronium bromide
**Muscle relaxant maintenance**	9.03	Assuming 2 amps of Pancuronium bromide.
**Inhalational anaesthetics (per minute)**	0.00	Assuming Isoflurane at a concentration of 50% and fresh gas flow of 3 litres per min.
**Consumables (all procedures)**	416.25	Gray et al. 2017 inflated to 2016/17 prices using PSSRU HCS inflation index.
**Additional consumables (on-pump procedures)**	104.06	Gray et al. 2017 inflated to 2016/17 prices using PSSRU HCS inflation index.
**Bypass machine time (per hour)**	15.21	Machine purchase cost plus maintenance contract annuitised assuming a machine lifespan of 7 years and assuming a 3.5% discount rate. Annual usage of machine is assumed to be eight hours per day, five days per week.
**Unit of red blood cells**	120.00	Assuming 294ml mean volume per unit.
**Unit of platelets**	193.15	Assuming 297ml mean volume per unit.
**Unit of fresh frozen plasma**	28.46	Assuming 246ml mean volume per unit.
**Unit of cryoprecipitate (pooled)**	177.57	Assuming 187ml mean volume per unit
**Laboratory issuing costs per blood product unit**	2.08	Campbell et al. 2015 inflated to 2016/17 prices using PSSRU HCS inflation index.
**Cell saver machine (per hour)**	1.44	Machine purchase cost plus maintenance contract annuitised assuming a machine lifespan of 7 years and assuming a 3.5% discount rate. Annual usage of machine is assumed to be eight hours per day, five days per week.
**Cell saver consumables**	104.06	Gray et al. 2017 inflated to 2016/17 prices using PSSRU HCS inflation index.
**Aprotinin (per dose)**	333.37	Assuming one-off cost for 6 million Kallikrein Inhibitor Units intravenously; 2012/13 cost inflated to 2016/17 cost year using HCHS Inflation Index.
Post-theatre Resource Use		
**Ventilator**	37.78	Dasta et al. 2005 inflated to 2016/17 prices using PSSRU HCS inflation index.
**Intra-aortic balloon pump plus pressure transducer (per insertion)**	582.75	Gray et al. 2017 inflated to 2016/17 prices using PSSRU HCS inflation index.
Inotrope drug days		
**Adrenaline**	12.75	Assuming average dose of 0.08 mcg/kg/min, 4mg in total volume 50ml with 5% Dextrose
**Noradrenaline**	5.20	Assuming average dose of 0.05 mcg/kg/min, 4mg in total volume 50ml with 5% Dextrose.
**Dobutamine**	3.98	Assuming average dose of 6 mcg/kg/min, 250mg in total volume 50ml with 5%
**Renal support therapy (per day)**	328.38	NHS reference cost – Renal - Haemodialysis for Acute Kidney Injury, 19 years and over (LE01A)
Haemofiltration (single dose)		
**Mannitol**	6.15	Assumes a one off dose of 0.2-2g/kg intravenously.
**Frusemide**	0.09	Assumes a one off dose of 20-50mg intravenously.
Treatment of sternal wound infection		
**Antibiotics for superficial SWI (per course)**	10.32	Assuming antibacterial protocol of 1g Flucloxacillin IV 4 times daily for 2 days; and either 2g Flucloxacillin IV once daily, 500mg Cefalexin 3 times daily, or 450mg Clindamycin 3 times daily for 5 days.
**Antibiotics for deep sternal wound infection (per course)**	106.23	Assuming antibacterial protocol of 500mg Meropenem and 600mg iv for 3 doses, then 600mg Teicoplanin IV once daily, then 3 times daily for 7 days.
**VAC Dressing (per episode)**	303.44	Gray et al. 2017 inflated to 2016/17 prices using PSSRU HCS inflation index. Includes VAC canister, dressing and machine and assumes three applications. Costs provided by trial participating hospital.
**Debridement procedures**	2984.82	Assumed to require two hours of theatre time and prophylactic antibiotics. Time costed using theatre and anaesthetic drug costs
**Surgical reconstruction**	5953.62	Assumed to require four hours of theatre time and prophylactic antibiotics. Time costed using theatre and anaesthetic drug costs
SAEs during index admission		

**Myocardial infarction**	880.06	NHS Reference Costs – Non-elective inpatient - Actual or Suspected Myocardial Infarction (weighted average across CC scores) (EB10), less inpatient bed days costed using average excess bed day cost for the same currency code (weighted average across CC scores).
**Cerebrovascular accident**	1195.47	NHS Reference Costs – Non-elective inpatient - Cerebrovascular Accident, Nervous System Infections or Encephalopathy (weighted average across CC scores) (AA22), less inpatient bed days costed using average excess bed day cost for the same currency codes (weighted average across CC scores).
**Further CABG**	6794.63	NHS Reference Costs – Non-elective inpatient – Complex / Major / Standard Coronary Artery Bypass Graft (weighted average across CC scores) (ED26/7/8), less inpatient bed days costed using average excess bed day cost for the same currency codes (weighted average across CC scores).
**Further PCI**	2079.82	NHS Reference Costs – Non-elective inpatient – Complex / Standard Percutaneous Transluminal Coronary Angioplasty (weighted average across CC scores) (EY40/1), less inpatient bed days costed using average excess bed day cost for the same currency codes (weighted average across CC scores).
**Cardiac catheterisation only**	1607.76	NHS Reference Costs – Non-elective inpatient - Complex / Standard Cardiac Catheterisation Angioplasty (weighted average across CC scores) (EY42/3), less inpatient bed days costed using average excess bed day cost for the same currency codes (weighted average across CC scores).
**Major bleed**	5953.62	Assumed to require surgery for investigation and to incur the same cost as sternal wound infection requiring reconstruction.
Inpatient bed days		
**Intensive Therapy Unit**	624.96	NHS Reference costs - Critical care - weighted average across all unventilated critical care service codes (CCU01-91) (Currency code: XC07Z).
**High Dependency Unit**	385.03	Gray et al. 2017 inflated to 2016/17 prices using PSSRU HCS inflation index.
**Cardiac Ward**	348.44	NHS Reference costs - Non Elective Inpatients Excess Bed Days - Complex / Major / Standard Coronary Artery Bypass Graft (weighted average across CC scores) (ED26/7/8)
Post-hospital discharge		
**Other hospital (per day)**	348.44	Assumed same bed day cost as for cardiac ward above.
**Nursing home (per day)**	158.29	Unit Costs of Health and Social Care 2017 (section 1.3).
**Rehabilitation unit (total cost)**	2922.06	Unit Costs of Health and Social Care 2014 (section 1.6) inflated to 2016/17 prices using the PSSRU HCS inflation index. Assumes an average stay of 33 days.

S Table 2: Sensitivity analysis showing mean costs for a "Per protocol" analysis

	**Year 1 Mean cost**		**Year 2 Mean cost**		**Year 3 Mean cost**		**Year 4 Mean cost**		**Year 5 Mean cost**		**Total at year 5**		
	SITA	BITA	SITA	BITA	SITA	BITA	SITA	BITA	SITA	BITA	SITA	BITA	BITA vs SITA Mean difference (95% CI, p value)
**Healthcare contacts**													
**GP visits**	238	227	165	159	156	148	149	137	142	137	851	809	-41.5 (-98.7, 15.7; 0.147)
**Nurse visits**	46	49	17	20	20	21	20	21	20	20	122	130	7.9 (-12.9, 28.7; 0.441)
**Outpatient clinic visits**	246	305	154	198	125	170	160	149	140	145	826	966	140.7 (35.2, 246.3; 0.011)
**Cardiac rehabilitation visits**	371	370	87	81	65	52	51	67	41	42	616	613	-3.1 (-172.8, 166.7; 0.971)
**Number of nights in hospital**	170	240	87	56	45	79	68	66	108	81	477	522	44.8 (-110.3, 199.9; 0.556)
**Medications**													
**Total medication**	33	33	39	41	40	44	41	46	41	44	193	208	14.4 (-1.0, 29.8; 0.066)
**Adverse event treatment**													
**Myocardial infarction**	16	11	3	6	6	11	7	8	9	9	41	44	2.8 (-24.5, 30.2; 0.833)
**Cerebrovascular accident**	27	12	12	14	11	8	7	11	21	17	79	62	-16.2 (-51.6, 19.1; 0.353)
**Further CABG**	0	8	0	0	0	8	0	0	0	0	0	15	15.2 (-4.1, 34.5; 0.118)
**Further PCI**	53	33	44	23	43	28	18	33	33	30	192	147	-44.8 (-128.9, 39.3; 0.283)
**Revascularisation with catheter**	4	14	16	20	28	13	19	13	2	15	69	74	5.5 (-33.1, 44.1; 0.772)
**Sternal wound problems**	90	325	1	0	0	0	0	1	0	0	91	326	234.4 (74.4, 394.5; 0.006)
**Major bleed**	34	50	0	0	0	0	0	1	0	0	34	51	17.1 (-32.0, 66.2; 0.479)
**Other AEs (cost of hospital stay only)**	446	505	188	155	141	152	161	195	233	205	1169	1211	42.5 (-299.7, 384.7; 0.800)
**Death (cost of hospital stay only)**	60	5	36	21	5	44	57	11	31	40	189	122	-66.8 (-161.1, 27.5; 0.157)
**n**	1494	1294	1494	1294	1494	1294	1494	1294	1494	1294	1494	1294	2788

S Table 3: Sensitivity analysis showing mean costs for a “available case” analysis

	Year 1 Mean cost		Year 2 Mean cost		Year 3 Mean cost		Year 4 Mean cost		Year 5 Mean cost		Total at year 5 Mean difference (95% CI, p value)		
	SITA	BITA	SITA	BITA	SITA	BITA	SITA	BITA	SITA	BITA	SITA	BITA	BITA vs SITA
**Healthcare contacts**													
**GP visits**	243	238	165	162	153	148	147	138	140	140	779	759	-19.4 (-61.3, 22.5; 0.350)
**Nurse visits**	46	50	17	21	21	21	21	23	20	22	117	130	11.9 (-7.4, 31.3; 0.217)
**Outpatient clinic visits**	247	317	153	173	128	166	158	150	144	148	774	859	122.9 (53.5, 192.2; 0.001)
**Cardiac rehabilitation visits**	463	476	89	85	68	46	54	75	41	43	696	717	9.6 (-173.2, 192.3; 0.915)
**Number of nights in hospital**	849	1029	247	203	237	203	234	235	260	242	1675	1704	74.7 (-165.0, 314.4; 0.528)
**Medications**													
**Total medication**	38	38	40	42	41	45	42	47	42	43	189	201	11.9 (-3.5, 27.3; 0.125)
**Adverse event treatment**													
**Myocardial infarction**	16	13	3	5	3	9	9	6	11	11	38	40	-1.6 (-20.9, 17.7; 0.866)
**Cerebrovascular accident**	20	11	10	13	12	5	6	11	24	18	65	51	-11.6 (-42.7, 19.4; 0.448)
**Further CABG**	0	7	0	0	0	7	0	0	0	0	0	15	13.9 (-4.1, 31.8; 0.125)
**Further PCI**	56	41	45	24	42	44	18	28	34	26	176	155	-30.3 (-97.7, 37.0; 0.363)
**Revascularisation with catheter**	3	19	14	20	24	14	17	10	1	16	54	76	24.3 (-18.1, 66.8; 0.250)
**Sternal wound problems**	104	289	1	0	0	0	0	1	0	0	105	265	184.4 (43.6, 325.2; 0.012)
**Major bleed**	35	25	0	0	0	0	0	1	0	0	31	26	-10.0 (-46.8, 26.8; 0.582)
**Other AEs (cost of hospital stay only)**	458	504	203	174	182	142	188	207	285	216	1184	1138	-73.5 (-365.2, 218.2; 0.610)
**Death (cost of hospital stay only)**	40	4	5	0	2	7	10	2	0	5	58	15	-33.9 (-97.0, 29.1; 0.279)
**n**	1444	1414	1435	1390	1376	1322	1269	1245	1196	1215	1029	1038	

S Table 4: Costs for subgroups: diabetes

	No history of diabetes			Insulin dependent diabetes			Non-insulin dependent diabetes		
	SITA	BITA	BITA vs SITA	SITA	BITA	BITA vs SITA	SITA	BITA	BITA vs SITA
**Total cost of index admission**	11775	12562	787 (-30.1, 1604.5; 0.058)	12458	14426	1968 (-154.6, 4090.4; 0.067)	12279	13047	768 (-468.2, 2005.1; 0.211)
**Total costs of discharge locations**	650	551	-100 (-486.3, 286.4; 0.599)	341	532	191 (-106.0, 487.0; 0.195)	371	548	177 (-275.6, 629.9; 0.427)
**GP visits**	830	799	-30 (-83.6, 22.8; 0.250)	943	987	44 (-164.3, 252.3; 0.664)	938	916	-22 (-143.5, 98.6; 0.705)
**Nurse visits**	117	126	9 (-9.7, 28.6; 0.320)	123	225	102 (-76.2, 279.9; 0.247)	141	129	-12 (-47.4, 23.6; 0.495)
**Outpatient clinic visits**	802	883	81 (-62.6, 225.6; 0.255)	1124	2114	990 (-326.9, 2306.8; 0.133)	824	865	41 (-107.8, 188.9; 0.576)
**Cardiac rehabilitation visits**	649	654	5 (-183.0, 193.0; 0.957)	300	362	62 (-207.3, 331.7; 0.627)	511	560	49 (-528.6, 626.7; 0.862)
**Number of nights in hospital**	454	451	-4 (-100.6, 93.0; 0.936)	468	575	107 (-390.2, 604.1; 0.658)	547	734	187 (-380.7, 754.8; 0.503)
**Total medication**	203	204	1 (-21.3, 23.6; 0.919)	134	216	81 (23.2, 139.2; 0.009)	173	216	43 (6.2, 80.1; 0.024)
**Myocardial infarction**	40	40	-0 (-32.7, 32.6; 0.996)	56	81	25 (-96.9, 146.6; 0.675)	33	32	-1 (-34.0, 32.2; 0.954)
**Cerebrovascular accident**	79	57	-22 (-56.7, 13.0; 0.209)	45	75	30 (-127.8, 187.6; 0.697)	69	52	-17 (-120.4, 86.3; 0.737)
**Further CABG**	0	17	17 (-5.0, 38.3; 0.126)	0	0	.	0	0	.
**Further PCI**	187	157	-30 (-107.2, 47.6; 0.436)	0	154	154 (-31.8, 339.6; 0.099)	250	214	-37 (-136.7, 63.6; 0.459)
**Revascularisation with catheter**	66	69	3 (-45.1, 51.4; 0.895)	14	60	47 (-42.6, 136.2; 0.288)	83	129	45 (-32.7, 123.2; 0.242)
**Sternal wound problems**	68	158	90 (-23.5, 204.2; 0.115)	307	1395	1088 (-288.2, 2465.1; 0.115)	206	507	301 (-63.2, 665.5; 0.101)
**Major bleed**	32	51	18 (-38.5, 75.2; 0.513)	0	0	.	42	23	-19 (-121.6, 82.9; 0.699)
**Other AEs (cost of hospital stay only)**	1140	1091	-50 (-381.0, 281.8; 0.761)	2019	2042	23 (-2647.5, 2694.0; 0.986)	1247	1333	86 (-636.3, 809.0; 0.807)
**Death (cost of hospital stay only)**	178	82	-96 (-195.2, 3.3; 0.057)	23	785	761 (-171.5, 1694.1; 0.104)	242	98	-143 (-341.9, 55.2; 0.149)
**Total cost at 5 years**	17269	17951	681 (-605.0, 1967.9; 0.285)	18355	24028	5673 (256.9, 11089.1; 0.041)	17957	19403	1447 (-438.1, 3331.3; 0.126)

Total cost of index admission includes the total cost of surgery (Time in theatre (minutes), duration related theatre costs and staff, duration related anaesthetic costs, time on bypass (minutes), and other surgery costs (consumables, blood products, aprotinin), post-operative costs (Ventilation time, Intra-aortic balloon pump, Inotropic support, Renal support therapy, Hemofiltration) and any in hospital adverse events (Myocardial Infarction, Cerebrovascular accident, Further CABG, Further PCI, Revascularisation with catheter, Major bleed, Other AEs (cost of hospital stay only), Death (cost of hospital stay only))

S Table 5: Costs for subgroups: age groups

	**Aged < 70 Yrs**			**Aged >= 70 Yrs**		
	SITA	BITA	BITA vs SITA	SITA	BITA	BITA vs SITA
**Total cost of index admission**	11653	12401	748 (114.4, 1381.9; 0.023)	12562	13780	1218 (-84.8, 2521.3; 0.065)
**Total costs of discharge locations**	380	429	49 (-99.4, 197.3; 0.502)	1126	887	-238 (-1351.7, 875.3; 0.661)
**GP visits**	846	862	16 (-28.3, 59.3; 0.471)	879	746	-133 (-266.4, 1.3; 0.052)
**Nurse visits**	108	122	14 (-5.6, 33.3; 0.154)	159	165	6 (-41.6, 53.9; 0.791)
**Outpatient clinic visits**	816	933	116 (-32.1, 264.9; 0.119)	838	1019	181 (-143.6, 506.5; 0.259)
**Cardiac rehabilitation visits**	568	567	-1 (-248.1, 245.7; 0.992)	707	767	60 (-228.4, 348.3; 0.670)
**Number of nights in hospital**	446	442	-4 (-118.9, 110.0; 0.936)	541	698	157 (-271.2, 584.8; 0.455)
**Total medication**	190	213	22 (1.9, 43.0; 0.033)	203	190	-13 (-40.8, 14.1; 0.324)
**Myocardial infarction**	39	47	9 (-14.2, 31.6; 0.443)	42	23	-19 (-54.7, 17.5; 0.297)
**Cerebrovascular accident**	58	41	-17 (-53.6, 20.1; 0.358)	121	102	-19 (-110.8, 72.3; 0.667)
**Revascularisation with catheter**	0	17	17 (-5.7, 40.0; 0.134)	0	0	.
**Further CABG**	222	179	-43 (-148.8, 63.0; 0.412)	101	133	32 (-49.7, 113.2; 0.427)
**Further PCI**	70	86	16 (-28.0, 60.1; 0.459)	58	61	3 (-65.8, 71.3; 0.935)
**Sternal wound problems**	112	314	202 (-8.3, 413.2; 0.059)	88	246	158 (-11.2, 327.7; 0.066)
**Major bleed**	26	20	-6 (-40.6, 28.7; 0.725)	50	106	56 (-90.0, 202.6; 0.433)
**Other AEs (cost of hospital stay only)**	874	1071	197 (-188.0, 582.1; 0.302)	2081	1533	-549 (-1274.2, 176.9; 0.131)
**Death (cost of hospital stay only)**	67	100	32 (-33.3, 98.1; 0.320)	486	208	-278 (-559.0, 3.1; 0.052)
**Total cost at 5 years**	16474	17842	1368 (640.7, 2095.1; 0.001)	20042	20666	623 (-1986.6, 3233.0; 0.624)

Total cost of index admission includes the total cost of surgery (Time in theatre (minutes), duration related theatre costs and staff, duration related anaesthetic costs, time on bypass (minutes), and other surgery costs (consumables, blood products, aprotinin), post-operative costs (Ventilation time, Intra-aortic balloon pump, Inotropic support, Renal support therapy, Hemofiltration) and any in hospital adverse events (Myocardial Infarction, Cerebrovascular accident, Further CABG, Further PCI, Revascularisation with catheter, Major bleed, Other AEs (cost of hospital stay only), Death (cost of hospital stay only))

S Table 6: Costs for subgroups: on/off pump

	Off-pump			On-pump		
	SITA	BITA	BITA vs SITA	SITA	BITA	BITA vs SITA
**Total cost of index admission**	11851	12018	167 (-863.9, 1197.9; 0.737)	12025	13509	1484 (884.9, 2082.5; 0.000)
**Total costs of discharge locations**	645	546	-99 (-335.3, 137.6; 0.393)	538	554	16 (-501.0, 532.9; 0.950)
**GP visits**	706	699	-7 (-41.0, 26.3; 0.650)	955	927	-28 (-94.4, 38.4; 0.391)
**Nurse visits**	141	147	6 (-17.7, 30.1; 0.594)	110	123	14 (-11.0, 38.5; 0.262)
**Outpatient clinic visits**	997	1194	197 (0.7, 393.4; 0.049)	707	788	81 (-11.7, 173.1; 0.084)
**Cardiac rehabilitation visits**	834	620	-214 (-422.4, -6.4; 0.044)	456	623	166 (-11.4, 344.1; 0.065)
**Number of nights in hospital**	462	471	8 (-98.5, 114.8; 0.874)	481	540	59 (-160.0, 278.6; 0.581)
**Total medication**	205	215	10 (-11.0, 30.6; 0.333)	187	202	15 (-5.5, 35.4; 0.143)
**Myocardial infarction**	53	45	-8 (-43.4, 27.4; 0.643)	31	39	8 (-10.9, 26.9; 0.389)
**Cerebrovascular accident**	87	61	-27 (-70.6, 17.6; 0.224)	68	56	-12 (-62.5, 37.9; 0.617)
**Revascularisation with catheter**	0	15	15 (-7.7, 38.3; 0.180)	0	11	11 (-12.1, 34.1; 0.335)
**Further CABG**	228	157	-70 (-196.0, 55.5; 0.257)	165	177	12 (-63.8, 87.9; 0.746)
**Further PCI**	73	98	26 (-38.4, 90.2; 0.411)	63	67	4 (-43.7, 51.2; 0.871)
**Sternal wound problems**	41	273	232 (23.8, 439.4; 0.031)	149	319	170 (-37.0, 376.6; 0.103)
**Major bleed**	52	47	-5 (-79.9, 70.7; 0.900)	20	40	20 (-42.1, 82.3; 0.511)
**Other AEs (cost of hospital stay only)**	1250	1223	-27 (-280.0, 225.3; 0.823)	1184	1188	4 (-463.2, 471.4; 0.986)
**Death (cost of hospital stay only)**	280	236	-44 (-290.1, 202.0; 0.712)	118	53	-65 (-141.8, 11.5; 0.092)
**Total cost at 5 years**	17905	18065	160 (-1181.9, 1501.2; 0.805)	17256	19214	1958 (706.5, 3209.5; 0.004)

Total cost of index admission includes the total cost of surgery (Time in theatre (minutes), duration related theatre costs and staff, duration related anaesthetic costs, time on bypass (minutes), and other surgery costs (consumables, blood products, aprotinin), post-operative costs (Ventilation time, Intra-aortic balloon pump, Inotropic support, Renal support therapy, Hemofiltration) and any in hospital adverse events (Myocardial Infarction, Cerebrovascular accident, Further CABG, Further PCI, Revascularisation with catheter, Major bleed, Other AEs (cost of hospital stay only), Death (cost of hospital stay only))

S Table 7: Costs for subgroups: baseline history of myocardial infarctions

	No prior myocardial infarction			Prior myocardial infarction		
	SITA	BITA	BITA vs SITA	SITA	BITA	BITA vs SITA
**Total cost of index admission**	11694	12501	807 (32.2, 1582.4; 0.042)	12167	13123	956 (227.9, 1684.2; 0.012)
**Total costs of discharge locations**	530	599	69 (-208.9, 346.7; 0.614)	654	476	-178 (-768.7, 412.4; 0.540)
**GP visits**	853	824	-28 (-88.6, 31.6; 0.338)	859	842	-16 (-75.4, 42.5; 0.570)
**Nurse visits**	122	130	8 (-10.8, 26.9; 0.387)	122	138	16 (-9.2, 41.3; 0.201)
**Outpatient clinic visits**	799	851	52 (-36.3, 140.1; 0.236)	852	1113	260 (49.8, 471.2; 0.017)
**Cardiac rehabilitation visits**	657	711	54 (-149.8, 257.9; 0.590)	541	482	-58 (-238.6, 121.6; 0.509)
**Number of nights in hospital**	473	507	34 (-127.0, 194.8; 0.667)	472	511	40 (-111.0, 190.0; 0.592)
**Total medication**	192	200	8 (-18.3, 34.0; 0.544)	196	217	21 (-11.4, 53.3; 0.193)
**Myocardial infarction**	43	32	-11 (-39.7, 17.7; 0.436)	35	54	19 (-14.8, 53.8; 0.253)
**Cerebrovascular accident**	88	47	-40 (-82.0, 1.2; 0.056)	60	73	13 (-34.0, 59.3; 0.581)
**Revascularisation with catheter**	0	11	11 (-11.7, 32.8; 0.338)	0	16	16 (-14.7, 46.4; 0.296)
**Further CABG**	219	174	-45 (-126.0, 36.2; 0.265)	150	156	6 (-114.4, 125.9; 0.922)
**Further PCI**	79	60	-19 (-59.5, 21.3; 0.340)	51	109	58 (-8.7, 124.2; 0.085)
**Sternal wound problems**	141	238	96 (-59.9, 252.0; 0.216)	59	385	326 (86.8, 565.2; 0.010)
**Major bleed**	35	48	13 (-50.6, 76.7; 0.677)	29	34	5 (-48.7, 58.8; 0.847)
**Other AEs (cost of hospital stay only)**	1119	1062	-58 (-436.6, 321.2; 0.756)	1315	1354	39 (-540.5, 618.5; 0.891)
**Death (cost of hospital stay only)**	179	95	-84 (-271.8, 103.6; 0.365)	185	178	-8 (-205.2, 189.9; 0.937)
**Total cost at 5 years**	17222	18088	866 (-151.5, 1883.2; 0.092)	17746	19260	1514 (8.9, 3019.0; 0.049)

Total cost of index admission includes the total cost of surgery (Time in theatre (minutes), duration related theatre costs and staff, duration related anaesthetic costs, time on bypass (minutes), and other surgery costs (consumables, blood products, aprotinin), post-operative costs (Ventilation time, Intra-aortic balloon pump, Inotropic support, Renal support therapy, Hemofiltration) and any in hospital adverse events (Myocardial Infarction, Cerebrovascular accident, Further CABG, Further PCI, Revascularisation with catheter, Major bleed, Other AEs (cost of hospital stay only), Death (cost of hospital stay only))

S Table 8: Costs for subgroups: NYHA class

	NYHA class I & II			NYHA class III & IV		
	SITA	BITA	BITA vs SITA	SITA	BITA	BITA vs SITA
**Total cost of index admission**	12022	12869	847 (174.1, 1519.6; 0.016)	11451	12394	943 (-50.7, 1937.5; 0.062)
**Total costs of discharge locations**	609	466	-142 (-394.1, 110.0; 0.256)	493	837	344 (-576.5, 1263.6; 0.448)
**GP visits**	891	875	-16 (-65.7, 33.0; 0.500)	717	681	-36 (-135.9, 63.9; 0.463)
**Nurse visits**	113	124	10 (-9.1, 29.6; 0.285)	153	166	13 (-26.8, 53.3; 0.500)
**Outpatient clinic visits**	786	840	54 (-51.8, 160.7; 0.301)	957	1357	400 (-87.9, 887.5; 0.103)
**Cardiac rehabilitation visits**	594	633	38 (-122.9, 199.9; 0.628)	650	572	-78 (-373.3, 217.9; 0.592)
**Number of nights in hospital**	482	436	-46 (-131.6, 40.3; 0.283)	437	763	327 (-166.6, 820.2; 0.184)
**Total medication**	197	208	10 (-9.8, 30.7; 0.298)	179	203	24 (-9.8, 58.7; 0.152)
**Myocardial infarction**	37	34	-3 (-27.4, 20.7; 0.777)	48	65	17 (-11.5, 46.5; 0.225)
**Cerebrovascular accident**	77	67	-10 (-46.5, 26.0; 0.567)	69	24	-45 (-97.6, 6.6; 0.084)
**Revascularisation with catheter**	0	16	16 (-5.4, 38.0; 0.134)	0	0	0 (., .; .)
**Further CABG**	186	168	-18 (-85.1, 48.5; 0.577)	186	162	-24 (-141.8, 94.7; 0.685)
**Further PCI**	53	67	14 (-22.2, 51.1; 0.424)	112	121	9 (-106.1, 123.4; 0.877)
**Sternal wound problems**	111	302	191 (-14.2, 396.1; 0.067)	84	277	193 (-78.3, 463.8; 0.155)
**Major bleed**	25	31	6 (-45.4, 56.8; 0.820)	61	84	23 (-92.9, 138.6; 0.687)
**Other AEs (cost of hospital stay only)**	1149	1048	-100 (-485.2, 284.8; 0.597)	1423	1694	271 (-351.2, 893.1; 0.377)
**Death (cost of hospital stay only)**	225	113	-112 (-269.3, 45.7; 0.156)	21	181	161 (-9.5, 331.2; 0.063)
**Total cost at 5 years**	17556	18296	740 (-246.3, 1726.0; 0.135)	17039	19582	2542 (375.6, 4708.6; 0.023)

Total cost of index admission includes the total cost of surgery (Time in theatre (minutes), duration related theatre costs and staff, duration related anaesthetic costs, time on bypass (minutes), and other surgery costs (consumables, blood products, aprotinin), post-operative costs (Ventilation time, Intra-aortic balloon pump, Inotropic support, Renal support therapy, Hemofiltration) and any in hospital adverse events (Myocardial Infarction, Cerebrovascular accident, Further CABG, Further PCI, Revascularisation with catheter, Major bleed, Other AEs (cost of hospital stay only), Death (cost of hospital stay only))

S Table 9: Costs for subgroups: CCS class

	CCS class 0, I, II			CCS class III, IVa/b/c		
	SITA	BITA	BITA vs SITA	SITA	BITA	BITA vs SITA
**Total cost of index admission**	12045	12825	779 (107.5, 1451.0; 0.025)	11572	12627	1055 (50.1, 2059.7; 0.040)
**Total costs of discharge locations**	498	468	-30 (-231.8, 171.6; 0.761)	779	726	-53 (-995.9, 889.2; 0.908)
**GP visits**	856	820	-36 (-91.0, 19.1; 0.201)	853	856	3 (-83.0, 89.4; 0.942)
**Nurse visits**	118	130	12 (-10.5, 33.6; 0.305)	130	140	10 (-16.6, 37.1; 0.453)
**Outpatient clinic visits**	775	788	13 (-100.4, 126.0; 0.825)	929	1321	392 (19.3, 764.2; 0.039)
**Cardiac rehabilitation visits**	639	640	1 (-155.8, 158.2; 0.988)	531	574	43 (-161.8, 247.8; 0.680)
**Number of nights in hospital**	478	436	-43 (-160.0, 74.4; 0.474)	458	669	211 (-119.5, 542.0; 0.210)
**Total medication**	194	199	5 (-19.8, 30.5; 0.678)	193	222	30 (-9.2, 68.5; 0.135)
**Myocardial infarction**	42	33	-10 (-36.8, 17.4; 0.484)	33	59	26 (-16.1, 68.4; 0.224)
**Cerebrovascular accident**	84	62	-21 (-64.9, 22.1; 0.334)	56	46	-10 (-63.0, 43.1; 0.712)
**Revascularisation with catheter**	0	18	18 (-6.9, 43.8; 0.154)	0	0	.
**Further CABG**	174	152	-22 (-95.6, 51.6; 0.557)	222	199	-23 (-149.7, 103.1; 0.718)
**Further PCI**	53	65	12 (-25.7, 50.5; 0.524)	98	110	12 (-60.3, 84.3; 0.745)
**Sternal wound problems**	117	330	213 (46.3, 380.5; 0.012)	79	223	144 (-15.4, 302.6; 0.077)
**Major bleed**	28	27	-1 (-52.6, 51.5; 0.982)	42	75	33 (-63.1, 129.0; 0.501)
**Other AEs (cost of hospital stay only)**	1231	1043	-188 (-572.5, 196.1; 0.337)	1144	1519	375 (-271.2, 1022.0; 0.255)
**Death (cost of hospital stay only)**	238	148	-90 (-243.5, 64.3; 0.254)	55	85	30 (-104.4, 165.0; 0.659)
**Total cost at 5 years**	17571	18185	614 (-497.7, 1725.8; 0.266)	17175	19452	2278 (97.8, 4457.7; 0.041)

Total cost of index admission includes the total cost of surgery (Time in theatre (minutes), duration related theatre costs and staff, duration related anaesthetic costs, time on bypass (minutes), and other surgery costs (consumables, blood products, aprotinin), post-operative costs (Ventilation time, Intra-aortic balloon pump, Inotropic support, Renal support therapy, Hemofiltration) and any in hospital adverse events (Myocardial Infarction, Cerebrovascular accident, Further CABG, Further PCI, Revascularisation with catheter, Major bleed, Other AEs (cost of hospital stay only), Death (cost of hospital stay only))

S Table 10: Costs for subgroups: by selected country

	UK			Poland			Australia		
	SITA	BITA	BITA vs SITA	SITA	BITA	BITA vs SITA	SITA	BITA	BITA vs SITA
**Total cost of index admission**	11809	12518	709 (53.8, 1364.3; 0.034)	11218	12329	1111 (478.1, 1744.0; 0.001)	15268	15772	504 (-1234.3, 2243.2; 0.568)
**Total costs of discharge locations**	538	475	-63 (-436.3, 309.5; 0.739)	871	881	10 (-191.9, 211.9; 0.923)	211	181	-30 (-238.9, 178.3; 0.775)
**GP visits**	697	700	3 (-38.4, 44.4; 0.887)	1328	1340	12 (-86.0, 110.4; 0.807)	1596	1353	-243 (-468.1, -18.1; 0.034)
**Nurse visits**	176	189	13 (-10.2, 35.2; 0.280)	15	20	5 (-5.3, 15.1; 0.346)	43	6	-36 (-108.3, 35.8; 0.322)
**Outpatient clinic visits**	891	1048	157 (-45.3, 359.5; 0.128)	695	872	177 (-14.6, 368.9; 0.070)	663	625	-38 (-168.8, 92.3; 0.564)
**Cardiac rehabilitation visits**	845	855	10 (-173.7, 193.3; 0.916)	126	111	-15 (-103.0, 72.7; 0.734)	322	368	46 (-166.7, 259.2; 0.666)
**Number of nights in hospital**	531	536	5 (-115.7, 126.1; 0.933)	381	258	-123 (-300.7, 53.7; 0.171)	280	326	46 (-174.2, 266.7; 0.679)
**Total medication**	206	221	15 (-12.9, 41.9; 0.298)	149	157	9 (-23.6, 40.9; 0.598)	123	139	16 (-21.2, 53.4; 0.396)
**Myocardial infarction**	51	50	-2 (-33.7, 30.3; 0.918)	7	15	8 (-16.9, 32.7; 0.533)	47	23	-24 (-102.3, 54.7; 0.551)
**Cerebrovascular accident**	76	61	-15 (-56.6, 27.4; 0.495)	57	24	-33 (-93.8, 27.7; 0.285)	43	147	103 (-89.0, 295.7; 0.290)
**Further CABG**	0	19	19 (-7.5, 45.5; 0.159)	0	0	.	0	0	.
**Further PCI**	226	175	-51 (-136.8, 35.5; 0.249)	72	106	34 (-59.9, 127.8; 0.478)	154	97	-57 (-271.6, 158.0; 0.603)
**Revascularisation with catheter**	91	95	4 (-43.7, 51.3; 0.875)	0	49	49 (2.4, 94.8; 0.039)	0	0	.
**Sternal wound problems**	126	296	170 (16.7, 324.1; 0.030)	72	184	112 (-102.8, 327.8; 0.305)	69	948	879 (-74.5, 1832.1; 0.071)
**Major bleed**	39	52	13 (-50.6, 75.9; 0.695)	34	0	-34 (-101.5, 34.2; 0.330)	0	0	.
**Other AEs (cost of hospital stay only)**	1525	1429	-96 (-578.2, 386.6; 0.697)	605	364	-241 (-501.3, 18.5; 0.069)	792	1766	974 (56.8, 1890.7; 0.038)
**Death (cost of hospital stay only)**	225	146	-78 (-234.5, 77.8; 0.325)	61	100	40 (-179.0, 258.5; 0.721)	267	16	-251 (-525.6, 22.7; 0.072)
**Total cost at 5 years**	18052	18864	813 (-306.3, 1931.6; 0.154)	15691	16811	1120 (130.3, 2109.3; 0.027)	19878	21767	1889 (-682.2, 4460.4; 0.149)

Total cost of index admission includes the total cost of surgery (Time in theatre (minutes), duration related theatre costs and staff, duration related anaesthetic costs, time on bypass (minutes), and other surgery costs (consumables, blood products, aprotinin), post-operative costs (Ventilation time, Intra-aortic balloon pump, Inotropic support, Renal support therapy, Hemofiltration) and any in hospital adverse events (Myocardial Infarction, Cerebrovascular accident, Further CABG, Further PCI, Revascularisation with catheter, Major bleed, Other AEs (cost of hospital stay only), Death (cost of hospital stay only))

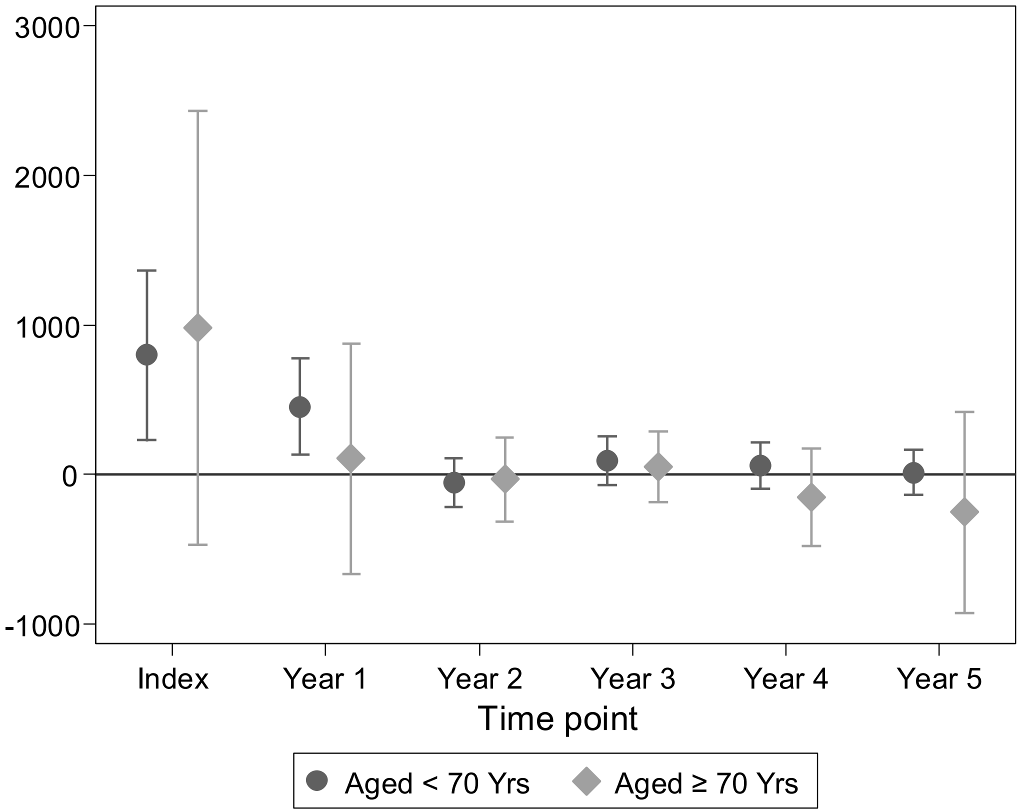

Figure S1 Differences in mean total costs (BITA vs SITA) by age groups over time

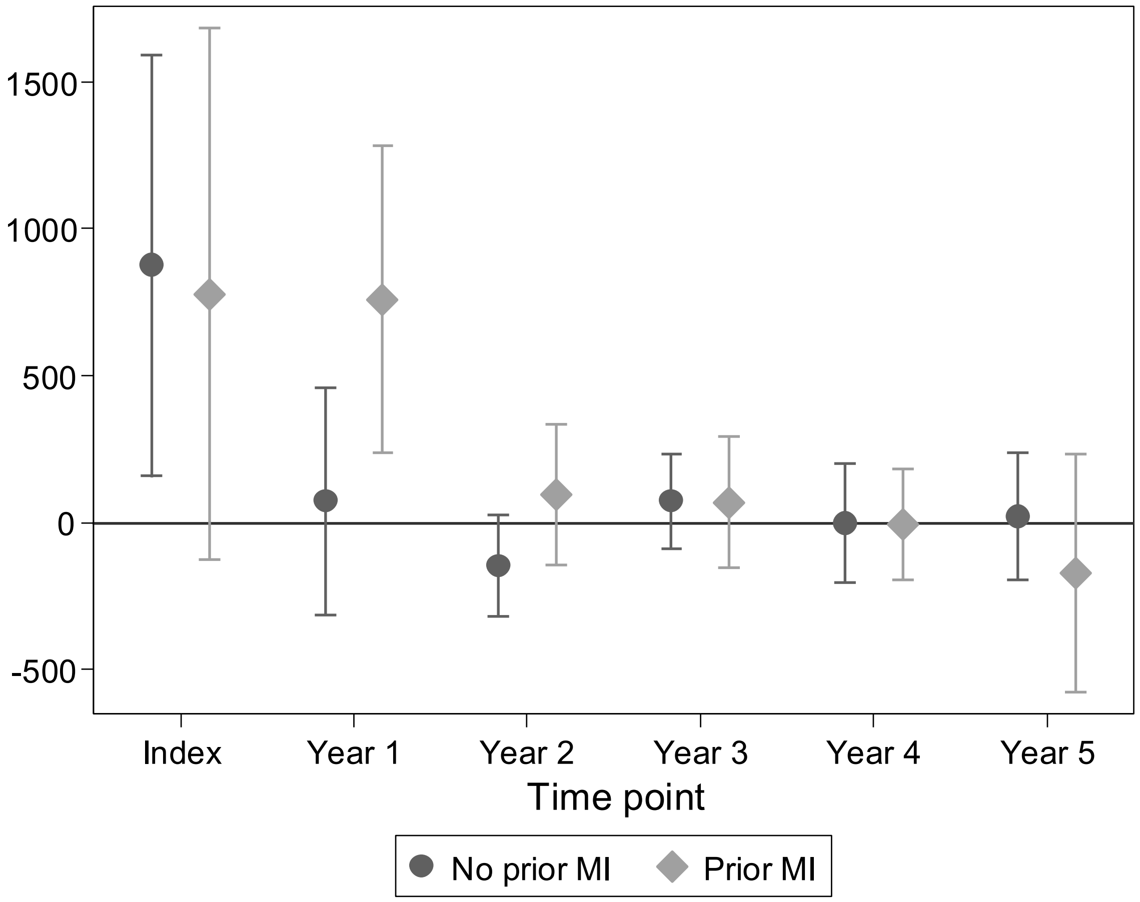

Figure S2 Differences in mean total costs (BITA vs SITA) by baseline prior history of myocardial infarction (MI) over time

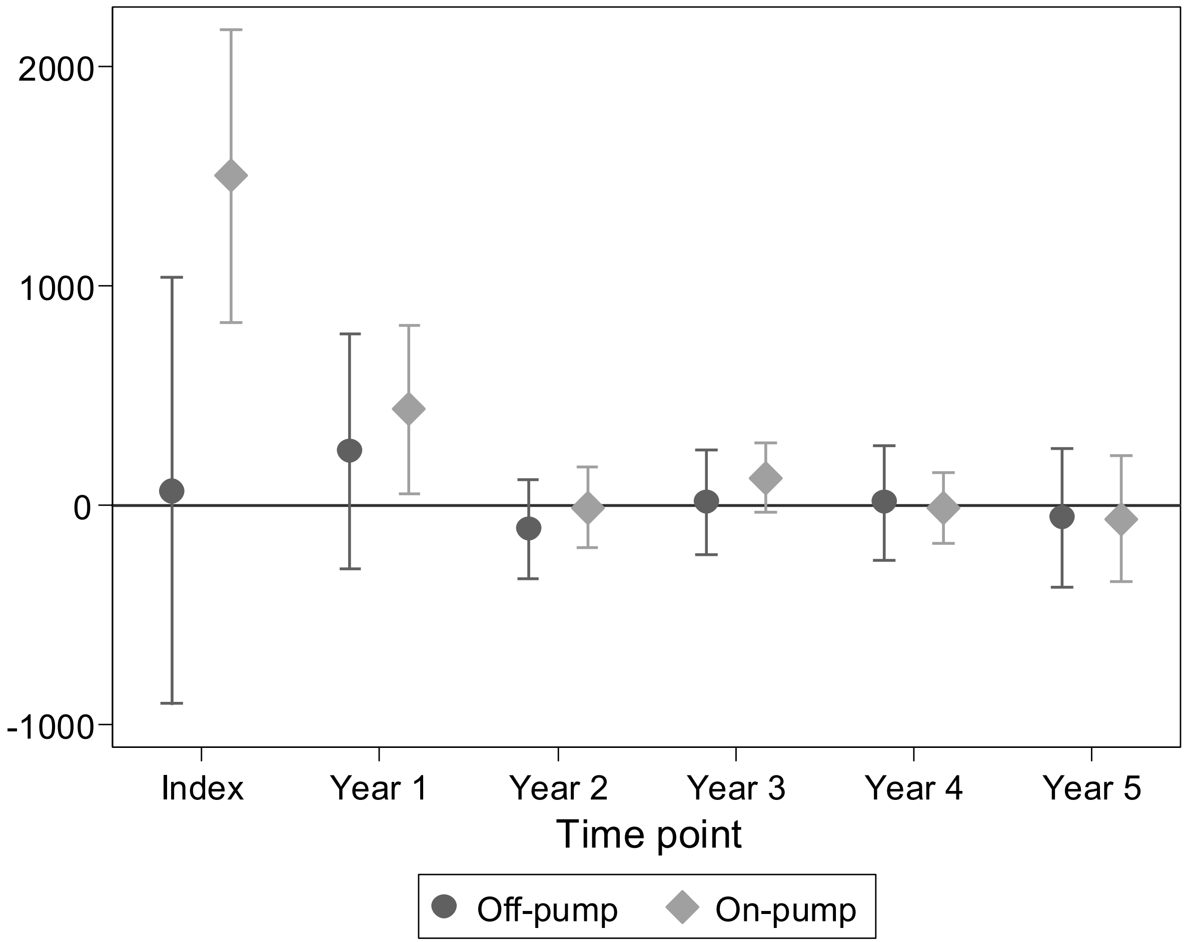

Figure S3 Differences in mean total costs (BITA vs SITA) by cardiopulmonary bypass over time

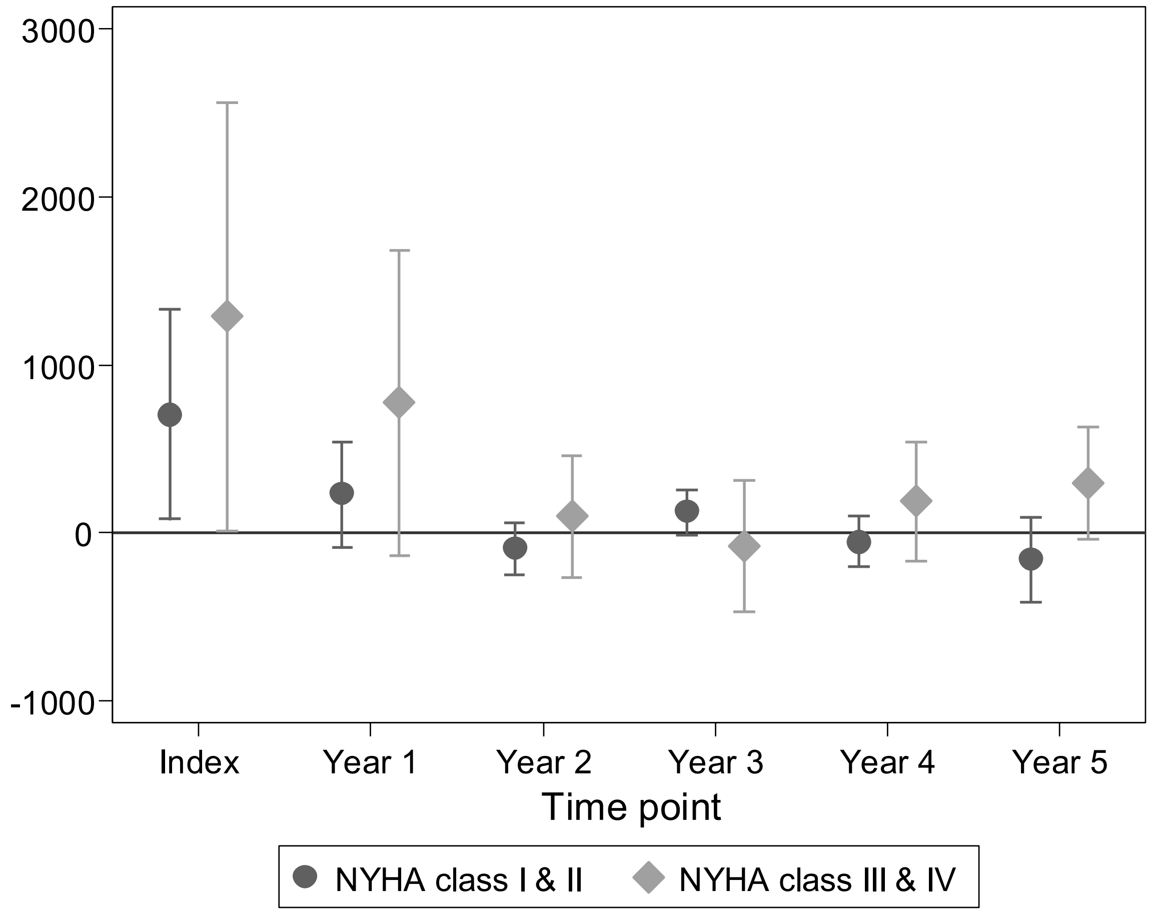

Figure S4 Differences in mean total costs (BITA vs SITA) by baseline New York Heart Association (NYHA) classification over time

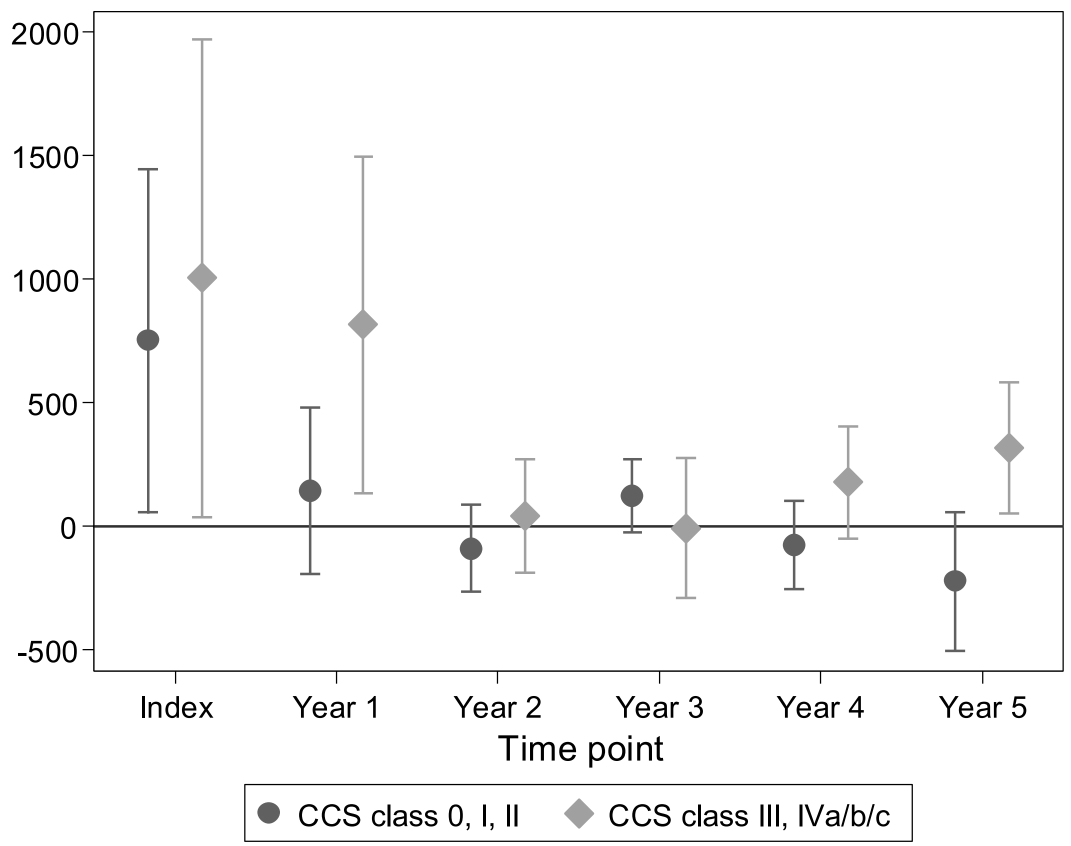

Figure 5 Differences in mean total costs (BITA vs SITA) by baseline Canadian Cardiovascular Society grading over time

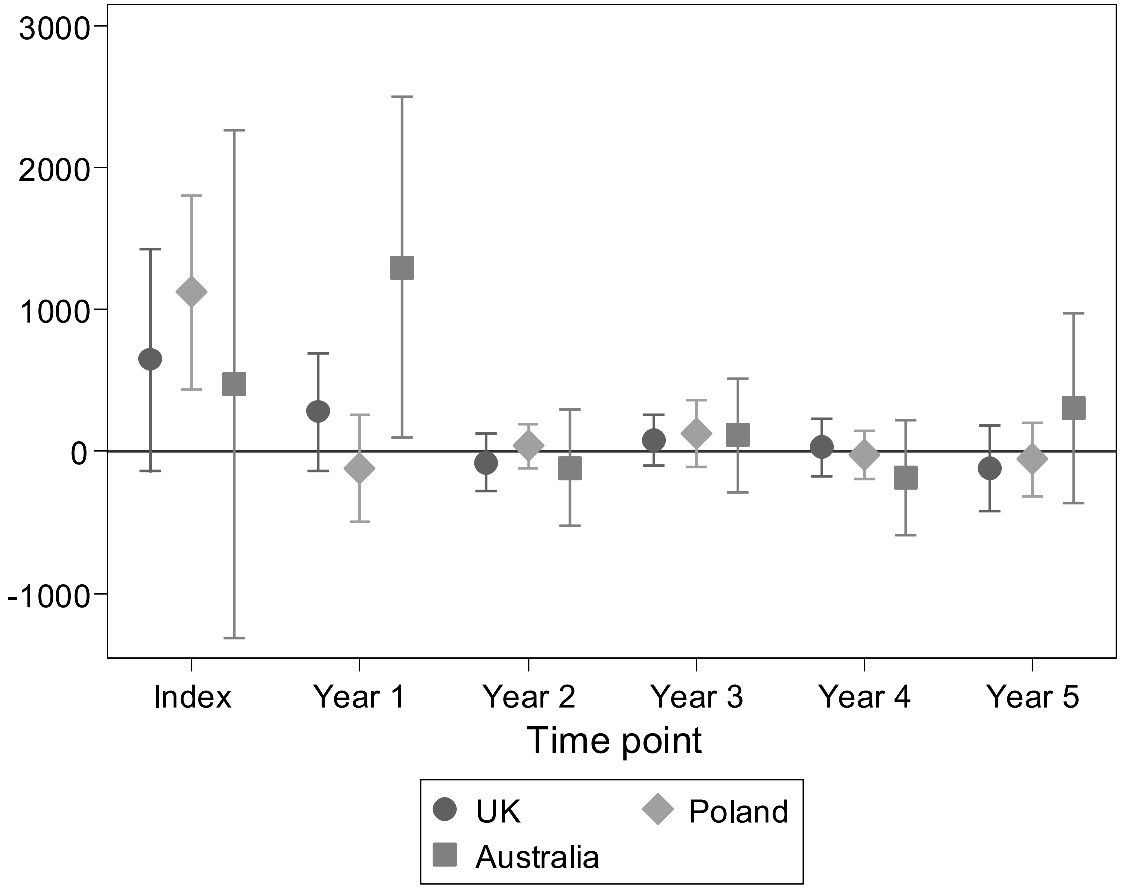

Figure S6 Differences in mean total costs (BITA vs SITA) by country over time

S Table 11: Mean and median total cost to five year follow-up

	SITA	BITA	BITA vs SITA
Mean	17480	18629	1149 (139, 2159; 0.027)
Median	14528	15609	1081 (584, 1577; 0.000)

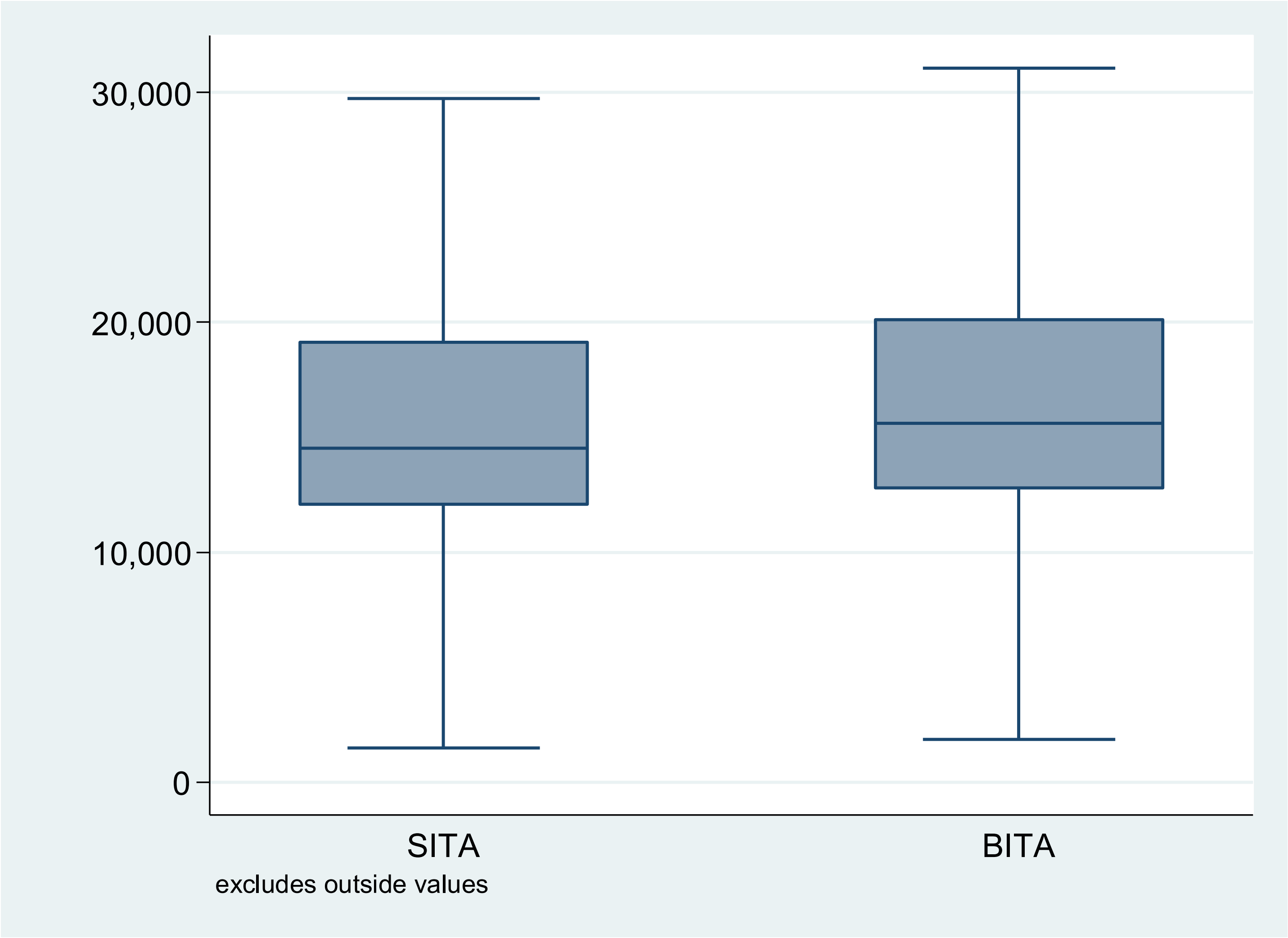

Figure S7 Boxplots of total costs to five year follow up (excluding outliers). Complete cases only.

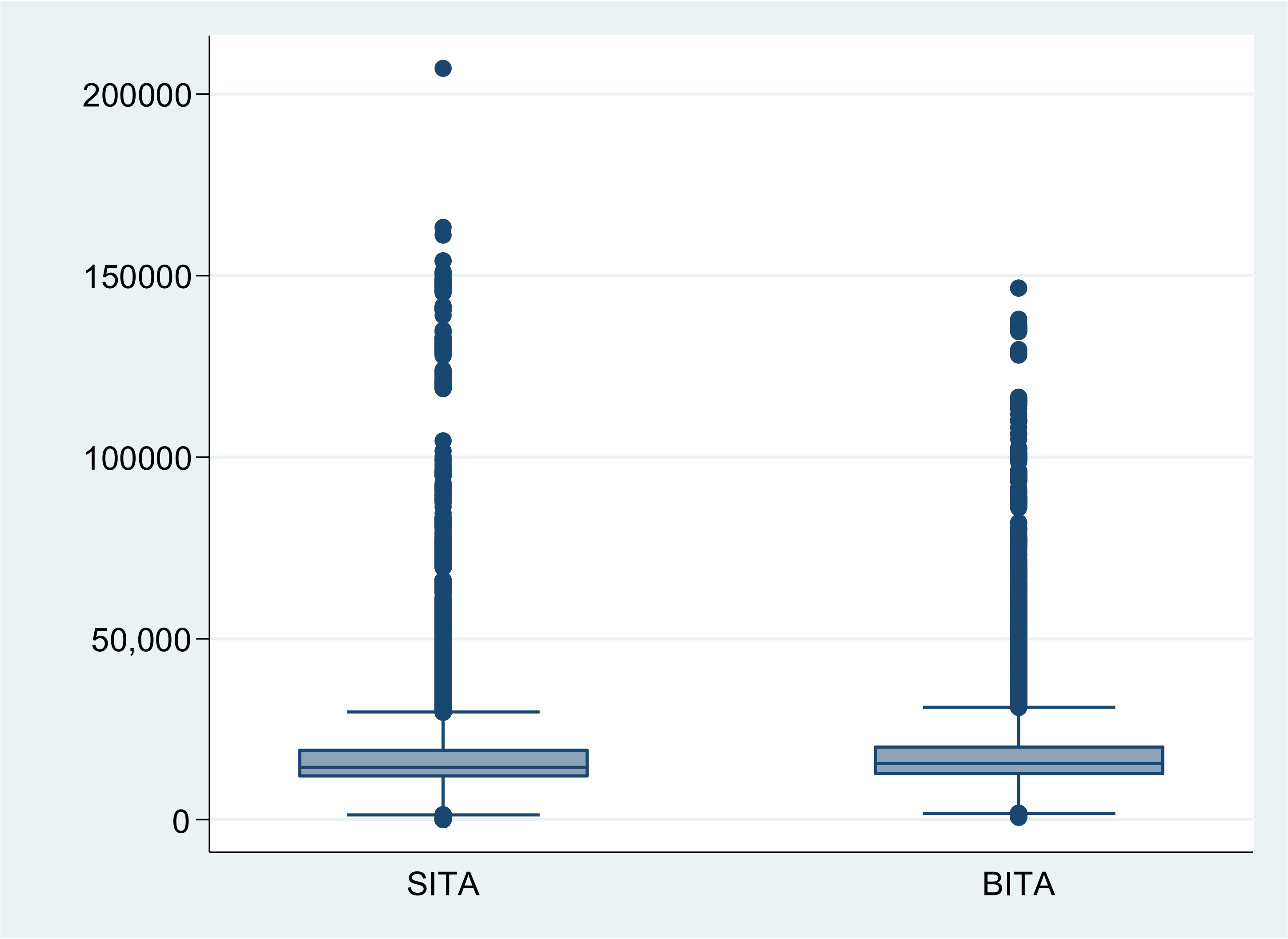

Figure S8 Boxplots of total costs to five year follow up (including outliers). Complete cases only.

S

S Table 12: Comparison of OLS and GLM estimates

	SITA	BITA	BITA vs SITA (p-value)
OLS	17,480	18,629	1149 (0.027)
GLM (Gamma, log link)	17,480	18,629	1149 (0.000)

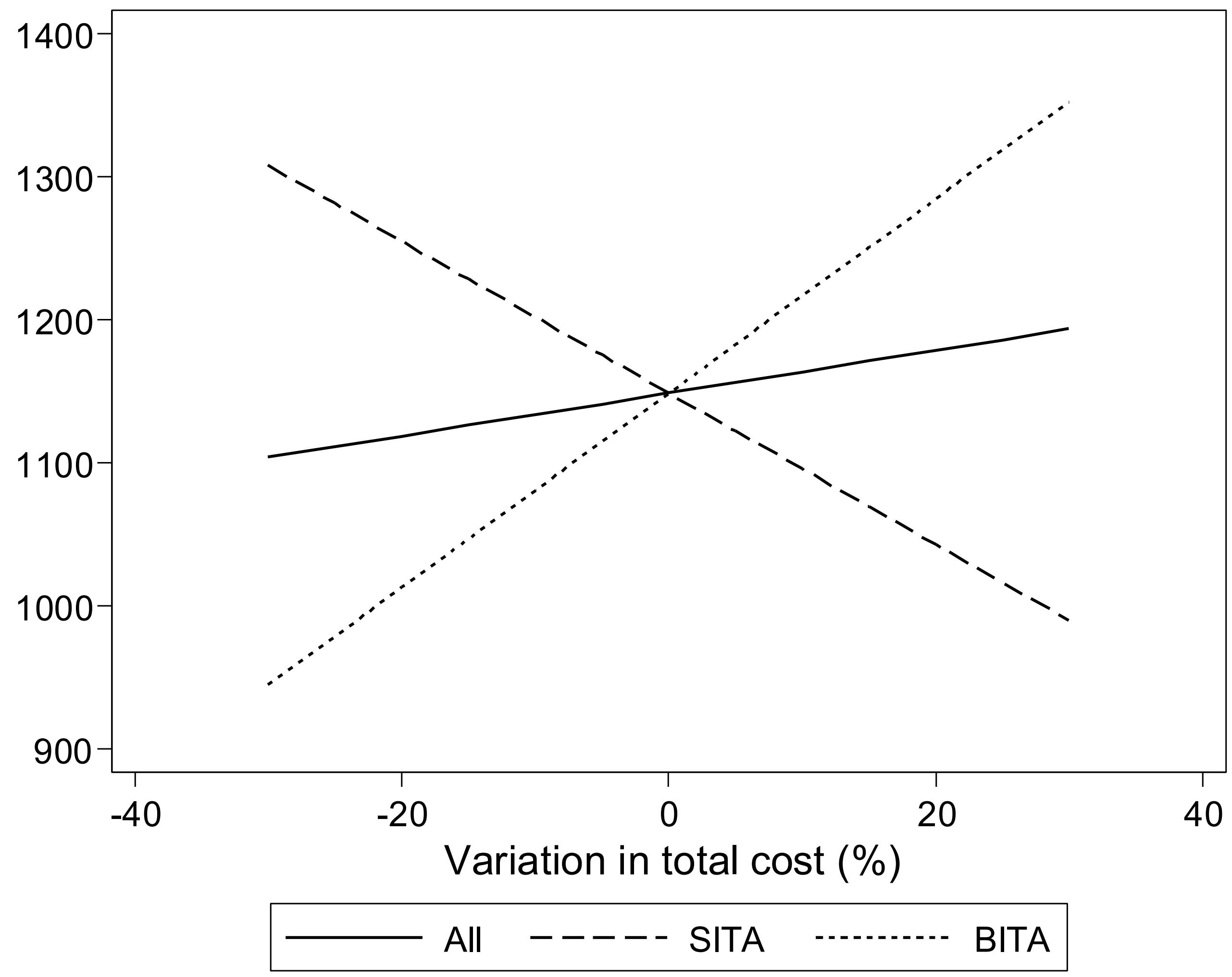

Figure S9 Sensitivity analysis assuming data are missing not at random. Imputed costs are varied between -30% and 30% at 5% point intervals.
